# The role of trust in the social heuristics hypothesis

**DOI:** 10.1371/journal.pone.0216329

**Published:** 2019-05-10

**Authors:** Andres Montealegre, William Jimenez-Leal

**Affiliations:** 1 Department of Psychology, Cornell University, Ithaca, NY, United States of America; 2 Department of Psychology, Universidad de los Andes, Bogotá, Colombia; Universidad Loyola Andalucia, SPAIN

## Abstract

According to the social heuristics hypothesis, people intuitively cooperate or defect depending on which behavior is beneficial in their interactions. If cooperation is beneficial, people intuitively cooperate, but if defection is beneficial, they intuitively defect. However, deliberation promotes defection. Here, we tested two novel predictions regarding the role of trust in the social heuristics hypothesis. First, whether trust promotes intuitive cooperation. Second, whether preferring to think intuitively or deliberatively moderates the effect of trust on cooperation. In addition, we examined whether deciding intuitively promotes cooperation, compared to deciding deliberatively. To evaluate these predictions, we conducted a lab study in Colombia and an online study in the United Kingdom (*N* = 1,066; one study was pre-registered). Unexpectedly, higher trust failed to promote intuitive cooperation, though higher trust promoted cooperation. In addition, preferring to think intuitively or deliberatively failed to moderate the effect of trust on cooperation, although preferring to think intuitively increased cooperation. Moreover, deciding intuitively failed to promote cooperation, and equivalence testing confirmed that this null result was explained by the absence of an effect, rather than a lack of statistical power (equivalence bounds: *d* = -0.26 and 0.26). An intuitive cooperation effect emerged when non-compliant participants were excluded, but this effect could be due to selection biases. Taken together, most results failed to support the social heuristics hypothesis. We conclude by discussing implications, future directions, and limitations. The materials, data, and code are available on the Open Science Framework (https://osf.io/939jv/).

## Introduction

Cooperation refers to actions that require an agent to pay a personal cost to benefit another individual or group [1]. Given that cooperating is costly for people and the benefits are distributed between group members, cooperation is puzzling. To understand cooperation, several researchers adopted a dual-process perspective in which decisions to cooperate or defect result from an interaction between intuitive and deliberative processes [2]. On one hand, we may cooperate intuitively but defect after reflection; on the other hand, we may defect intuitively but cooperate after reflection. In support of intuitive cooperation [3], faster decisions are more cooperative [2]. Moreover, promoting intuitive decisions through time constraints (i.e., deciding quickly) [2, 4–7], induction procedures (i.e., encouraging reliance on intuition) [2, 8], and cognitive load (i.e., engaging in an effortful task while deciding) increases cooperation [9]. Yet, some replication studies have failed to find evidence of intuitive cooperation [10].

To explain these inconsistent results, the social heuristics hypothesis (SHH) proposes that people intuitively cooperate or defect depending on which behavior is beneficial in their interactions [4, 7, 11]. If cooperation is beneficial, people intuitively cooperate, but if defection is beneficial, they intuitively defect. Though intuition is influenced by previous experience, deliberation promotes behaviors that maximize payoffs (here, defection). The SHH also argues that experience with experiments changes people’s intuition towards defection. Therefore, according to the SHH, intuition promotes cooperation, but non-cooperative interactions or experience with experiments reduce this effect. Supporting these predictions, an analysis of 15 studies showed that time pressure increases cooperation, whereas experience with experiments reduces this tendency [4]. A study also found that non-cooperative interactions reduces intuitive cooperation [2]. More generally, a meta-analysis of 67 studies found that promoting intuition, rather than deliberation, increases cooperation in settings in which noncooperation always maximizes payoffs [11]. However, high-powered replications have failed to replicate intuitive cooperation effects. In particular, a registered replication report found no effect of deciding quickly (versus slowly), even when excluding participants who had experience with experiments [12], and another replication attempt found no effect of encouraging intuitive decisions (compared to encouraging deliberative decisions) [13]. Though there are methodological concerns with many of these studies [12, 14, 15], two recent attempts (one pre-registered) that addressed most of these concerns found evidence of intuitive cooperation [16, 17]. Given the mixed evidence, the validity of the SHH remains contested.

Here, we evaluate the predictive power of the SHH by testing its predictions regarding trust and intuitive cooperation. Trust refers to a “a psychological state comprising the intention to accept vulnerability based upon the positive expectations of the intentions or behavior of another (p. 395)” [18]. According to the SHH, cooperative interactions promote trust towards interaction partners, whereas non-cooperative interactions promote distrust towards them [19]. In turn, deciding intuitively promotes cooperation among trusting people, whereas it has less of an effect (or no effect) among distrusting people [7, 20]. As predicted by the SHH, promoting intuition increases cooperation among trusting and naïve participants [7], but not among untrusting and naïve participants [20]. Nonetheless, the evidence is correlational and consistent with multiple possibilities. One possibility is that trust in everyday interactions influences intuitive cooperation (the one favored by the SHH). However, another possibility is that people report that they are trusting after observing themselves cooperating intuitively [21]. Therefore, whether trust has a causal impact on intuitive cooperation remains unclear.

The SHH also suggests that the effect of trust on cooperation depends on people’s reliance on intuition and deliberation (that is, an individual differences approach to the influence of cognitive processes). The SHH predicts that the effect of trust on cooperation is greater among people that tend to rely on intuition or that do not tend to rely on deliberation [19]. As expected by the SHH, exposing people to environments that support or undermine cooperation influences their prosocial behavior in the expected direction (increases or decreases it, respectively), and the effect is more pronounced among those that tend to rely on heuristics [19]. Yet, the evidence is limited, given that the measure of reliance on heuristics (i.e., Cognitive Reflection Test) is a good measure of reflection but not of intuition [22]. Thus, whether trust has a greater impact on cooperation among people that tend to follow their intuitions, tend to avoid deliberating, or both, remains untested.

### Present studies

We tested two predictions regarding the role of trust in intuitive cooperation. First, we evaluated the causal effect of trust on intuitive cooperation. Based on the SHH, we expected that promoting high trust, compared to low trust, would increase cooperation to a greater degree when participants decide intuitively rather than deliberatively (hypothesis 1). Second, we tested whether the impact of trust on cooperation is moderated by information processing preferences. We anticipated trust to have a greater effect on cooperation among people that like an intuitive processing style, dislike a deliberative processing style, or both (hypothesis 2). We also examined the main prediction of the SHH: We expected promoting intuition to increase cooperation, relative to promoting deliberation (hypothesis 3).

To evaluate these hypotheses, we ran two studies. Study 1 was conducted in the lab in Colombia, and Study 2 was conducted online in the United Kingdom. In both studies, we manipulated trust (high vs. low) and cognitive processes (intuition vs. deliberation). To manipulate trust, participants recalled an experience in which trusting other people led to either positive or negative outcomes in order to induce high or low trust, respectively. To manipulate cognitive processes, participants decided under time pressure or time delay (Study 1), or were encouraged to decide intuitively or deliberatively (Study 2). Preferences for processing information intuitively or deliberatively were measured using the Rational-Experiential Inventory in Study 1 [23].

Contrary to expectations, the results did not support our predictions. Inducing trust failed to increase intuitive cooperation (hypothesis 1), though trust increased cooperation. Also, preferring to process information intuitively or deliberatively failed to moderate the effect of trust on cooperation (hypothesis 2), although preferring to process information intuitively increased cooperation. Moreover, promoting intuition failed to increase cooperation (hypothesis 3), and equivalence testing confirmed that this null result was due to the absence of an effect, rather than a lack of statistical power. An intuitive cooperation effect appeared when non-compliant participants were excluded, but this result could be due to selection biases [12]. Our findings are limited in various respects: The trust induction was not that robust or strong, and there were issues of non-compliance, non-comprehension, and experience with previous studies. Nonetheless, excluding non-comprehending and experienced participants did not change the results considerably. In all studies, we report how we determined our sample sizes, all data exclusions, all manipulations, and all measures [24]. We conducted another study comparing the predictive power of self-report and behavioral measures of trust on intuitive cooperation that is not included in the paper given that it is underpowered (see S1 Appendix for design and results). All materials, data, and code are available on the Open Science Framework (https://osf.io/939jv/).

## Study 1

In Study 1, we tested the causal effect of trust on intuitive cooperation. Participants were exposed to a trust induction (high vs. low) before a one-shot public goods game under time pressure or time delay to promote intuition or deliberation, respectively. We also measured participants’ preferences to process information intuitively or deliberatively. We expected that inducing high trust, relative to low trust, would have a greater effect on cooperation under time pressure than under time delay (hypothesis 1). We anticipated a greater impact of trust on cooperation among people who like an intuitive processing style, dislike a deliberative processing style, or both (hypothesis 2). Finally, we expected that time pressure would increase cooperation, compared to time delay (hypothesis 3).

### Methods

287 participants (164 men, 121 women, and 2 other, *M*_age_ = 21.89, *SD* = 3.21) participated in the study in exchange for $6,000 Colombian Pesos. They could gain $4,000 to $20,000 Colombian Pesos more if they were selected in a lottery. We aimed for 300 participants (75 per condition), and ended up with 303 participants. We excluded participants that did not reach the end of the survey or that did not select a contribution in the game. We also excluded the second observation of someone who participated twice and an observation recorded after the study concluded. Studies were approved by the ethics committee of the Psychology Department at Universidad de los Andes. All participants provided their consent in Qualtrics. We conducted the study at a university in Colombia, and not online, to target a population with little experience with similar studies. MTurk participants have become too experienced with similar experiments, and the intuitive cooperation effect has declined such that, if any effect remains, it is difficult to detect [4]. The study was conducted in Spanish.

Participants were exposed to a trust induction and played a one-shot public goods game under time constraints. The study had a 2 (trust induction: high vs. low) × 2 (time constraint manipulation: time pressure vs. time delay) between-subjects design. In the trust induction, adapted from a manipulation of cognitive processes [2, 25], participants wrote a paragraph describing a moment in their lives in which trusting other people led to positive or negative consequences in order to induce high or low trust, respectively. To reduce potential demand effects, we presented it as an exercise to check that participants were concentrated on the task [26]. To check if the induction worked, we asked participants to report their experienced trust in the game, and we introduced questions about emotions to conceal the purpose of the question. Responses were measured on a scale ranging from 0 *nothing* to 9 *very much* (coded from 1 to 10). To promote intuition or deliberation, participants decided under time pressure or time delay, respectively [2]. Time pressure participants were asked to answer as fast as possible, in less than 10 seconds (a timer counting from 10 to 0 was presented on the decision screen), whereas time delay participants were asked to carefully consider their decision and wait a minimum of 10 seconds (a timer counting from 0 until participants responded was presented on the decision screen). Decision times were recorded to check if participants complied with the instructions.

To measure cooperation, participants played a four-player one-shot anonymous public goods game. They choose how much to contribute to the common pool from their endowment ($0-$8,000 Colombian Pesos, using a slider that started in the middle). Their contributions were multiplied by two and divided equally among players (i.e., the game had a marginal return of 0.5). We used a one-shot game given that there is no incentive to cooperate under these conditions (i.e., the payoff-maximizing behavior is defection) and, thus, the SHH predicts that deliberation promotes defection [4]. The game involved real monetary incentives, a random payment scheme (one out of every four players in a session was paid the amount won), and no deception. The sessions were conducted in groups of 8 or 12 participants to guarantee that they could not identify others in their group. Decisions were made on a computer in an individual cubicle. After the game, we measured comprehension of the task using two 9-option multiple choice questions. To measure preferences for information processing, we administered the 10-item Rational-Experiential Inventory [23], composed of two scales: Need for Cognition (5-items) and Faith in Intuition (5-items). The Need for Cognition scale measures a tendency to process information through effortful thinking (that is, a preference for deliberative processing), whereas the Faith in Intuition scale measures a tendency to process information by following one’s intuition (that is, a preference for intuitive processing). Responses were measured on a scale from 1 *completely false* to 5 *completely true*. We also measured experience with similar studies, age, and gender, among other questions (see S1 Fig for summary of all tasks). At least one-week before the study, participants answered a survey including trust questions along with other filler items. Our protocol and materials were based on the time constraint registered replication report [12].

### Results

For all analyses, we adopted a 5% alpha level and, unless otherwise stated, we ran ordinary least squares regressions. We use the term *significant* to mean statistically significant, not practically important [27]. Regression tables can be found in S1 Table [28]. We also calculated 95% confidence intervals to examine the set of plausible values for estimates [29].

Before testing the hypotheses, we checked the impact of the manipulations, and examined comprehension of the task, preexisting trust, and experience with similar studies. As expected, participants in the high trust condition felt significantly more trust (*M* = 6.19, *SD* = 2.54, *n* = 145) than those in the low trust condition (*M* = 5.37, *SD* = 2.55, *n* = 142), Welch’s *t*-test: *t*(284.8) = -2.71, *p* = .007, *M*_*diff*_ = -0.81, 95% CI [-1.4, -0.22], suggesting that the induction had the expected effect, though the means of the high and low trust conditions were not particularly high or low, respectively. Also as predicted, participants responded faster under time pressure (*M* = 15.47, *SD* = 8.37, *Mdn* = 12.95, *n* = 145) than under time delay (*M* = 33.99, *SD* = 31.08, *Mdn =* 26.16, *n* = 142). Given that decision times were right-skewed, we applied a log_10_ transformation (see Fig A in S2 Fig) and found significant differences between log_10_ decision times under time pressure and time delay, Welch’s *t*-test: *t*(246.31) = 9.23, *p* < .001. Nonetheless, the median decision time under time pressure was higher than 10 seconds, and only 26% of participants complied under time pressure, compared to 90% under time delay. Additionally, only 56% of participants passed both comprehension checks. In light of the high non-compliance and non-comprehension, we conducted exploratory analyses excluding these participants. However, these analyses may bias estimates of causal effects given that they condition on posttreatment variables [30]. We examined levels of trust before the study and found relatively high levels, *M* = 7.09, *SD* = 1.84, *n* = 246. Most participants (79%) had no experience with similar studies.

#### Hypothesis 1

**Fig 1:**
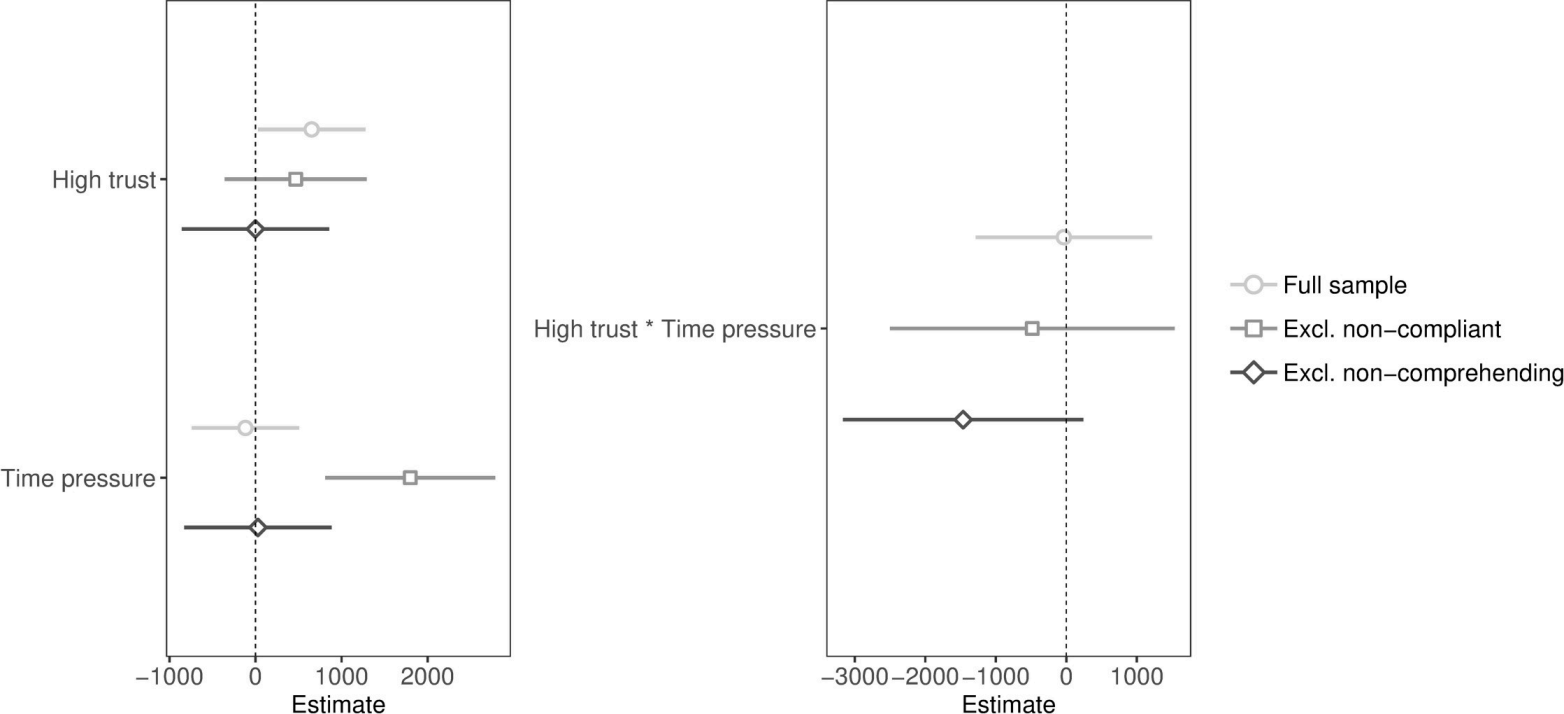
Unstandardized coefficients and 95% confidence intervals of public goods game contributions predicted by high trust and time pressure (with and without interaction and exclusions). Coefficients of regression with main effects are presented on the left and coefficients of regression with interaction and simple effects are presented on the right (simple effects are not included in the plot).

We expected that promoting high trust, compared to low trust, would have a greater effect on cooperation under time pressure than under time delay (see Table 1 for descriptive statistics and Fig 1 for regression plots). We first entered main effects: high trust (1 = high trust, 0 = low trust) and time pressure (1 = time pressure, 0 = time delay), and then added the interaction along with simple effects. Contrary to our hypothesis, we found no significant interaction between high trust and time pressure (*p* = .96), even when excluding non-compliant (*p* = .64) or non-comprehending participants (*p* = .09). The confidence interval of the sample excluding non-comprehending participants indicated that the majority of plausible values for the interaction were negative (i.e., high trust increases cooperation under time delay, but decreases cooperation under time pressure). We found a significant main effect of high trust (full sample: *p* = .041), that became non-significant after exclusions (excluding non-compliant: *p* = .27; excluding non-comprehending: *p* = .997), and a null main effect of time pressure (full sample: *p* = .71; excluding non-comprehending: *p* = .95), that became significant after excluding non-compliant participants (*p* < .001).

**Table 1:** Public goods game contributions, compliance, and comprehension depending on the condition.

Trust	Time constraint	*n*	*M*_contribution_	*SD*_contribution_	*n*_complied_	*n*_comprehend_
High	Time pressure	70	5,349.80	2,760.94	23	38
	Time delay	75	5,484.69	2,729.20	65	45
Low	Time pressure	75	4,714.92	2,496.45	14	46
	Time delay	67	4,814.66	2,774.60	63	32

Descriptive statistics of contributions are calculated in the full sample.

#### Hypothesis 2

We anticipated trust to have a greater effect on cooperation among participants who like an intuitive processing style, dislike a deliberative processing style, or both. The scales had questionable internal consistency: Faith in Intuition (α = 0.62) and Need for Cognition (α = 0.67). We first entered main effects (high trust, time pressure, and Faith in Intuition or Need for Cognition) and then added interactions along with simple effects. Against our hypothesis regarding intuitive processing (see Fig B in S2 Fig), we found no significant interaction between high trust and Faith in Intuition (full sample: *p* = .31; excluding non-compliant: *p* = .49; excluding non-comprehending: *p* = .34). We found a significant main effect of Faith in Intuition (full sample: *p* = .012), that was not significant after exclusions (excluding non-compliant: *p* = .057; excluding non-comprehending: *p* = .069), though the confidence intervals indicated that the majority of plausible values for the effect were positive. We also found a non-significant main effect of high trust (full sample: *p* = .055; excluding non-compliant: *p* = .29; excluding non-comprehending: *p* = .94). Still, the confidence interval of the full sample indicated that the majority of likely values for the effect were positive. Contrary to our hypothesis concerning deliberative processing (see Fig C in S2 Fig), we found no significant interaction between high trust and Need for Cognition (full sample: *p* = .49; excluding non-compliant: *p* = .77; excluding non-comprehending: *p* = .28). We found a significant main effect of high trust (full sample: *p* = .035), that became non-significant after exclusions (excluding non-compliant: *p* = .27; excluding non-comprehending: *p* = .99). We also found no significant main effect of Need for Cognition (full sample: *p* = .17; excluding non-compliant: *p* = .72; excluding non-comprehending: *p* = .49), though the confidence interval of the full sample indicated that the majority of plausible values for the effect were positive. If responses to the scales were affected by the manipulations, then there could be post-treatment bias but, unfortunately, this problem cannot be diagnosed empirically [30].

#### Hypothesis 3

**Fig 2:**
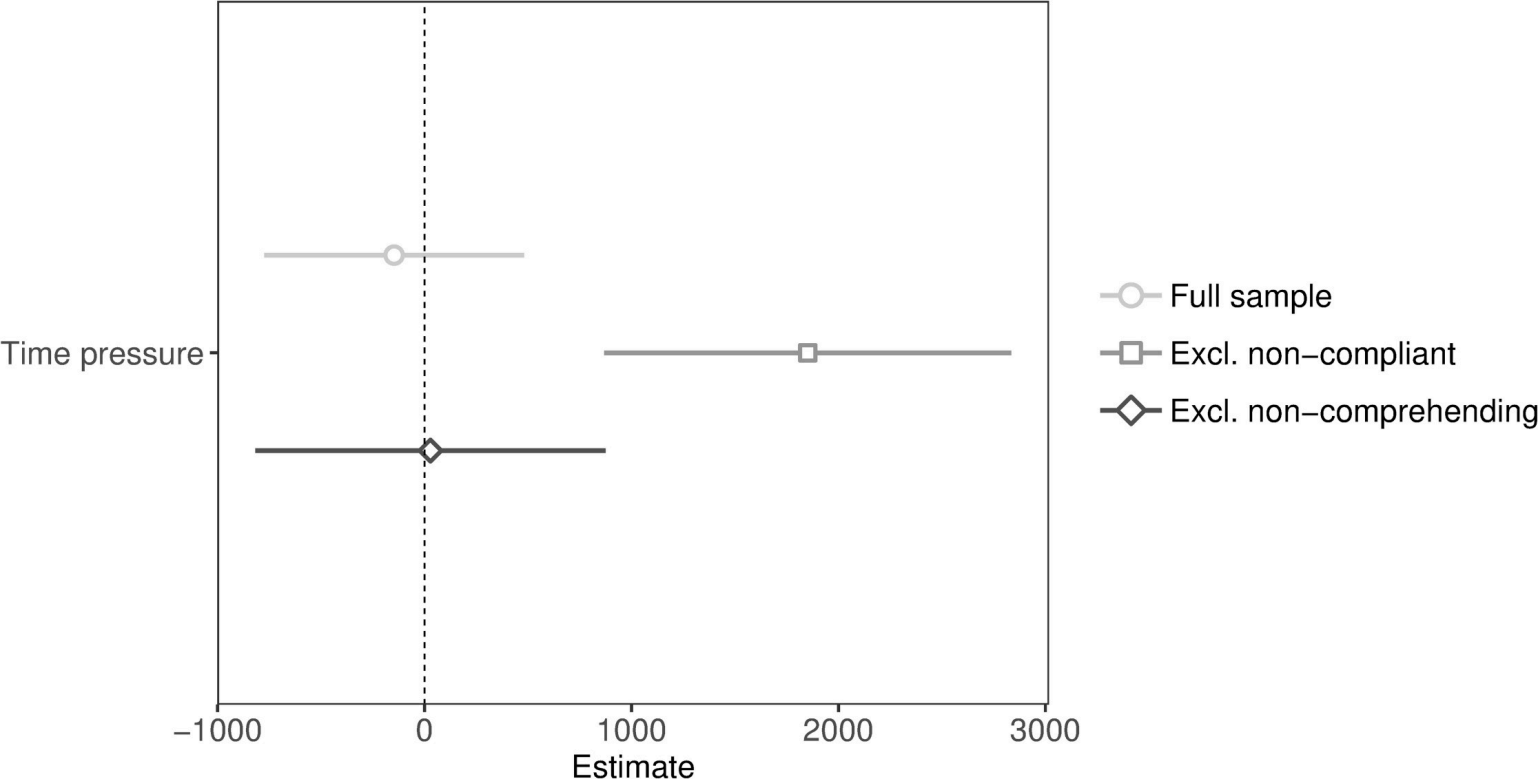
Unstandardized coefficients and 95% confidence intervals of public goods game contributions predicted by time pressure (with and without exclusions).

We expected that time pressure would increase cooperation, compared to time delay. Unexpectedly, we found no significant effect of time pressure (full sample: *p* = .65), even when excluding non-comprehending participants (*p* = .95; see Fig 2 for regression plot). Given that null results can be due to the absence of an effect or lack of statistical power, we tested for the absence of an effect using equivalence tests (in the full sample) [31–33]. We ran two one-sided tests to check for equivalence. We determined the smallest effect size of interest using the “small telescopes” approach (i.e., setting the smallest effect size of interest to the effect that a study had 33% power to detect) [31, 34]. We chose the intuitive cooperation effect in Rand and collaborators’ Study 7 from their 2012 paper given the theoretical similitude of the result, *d* = 0.40, 95% CI [0.06, 0.73], *n* = 153 (non-compliant participants were excluded) [2]. The study had 33% power to detect an effect of *d* = 0.26, and we therefore used this effect to determine the equivalence bounds. We used the two one-sided tests procedure to test the null hypothesis that the effect falls outside the equivalence bounds, i.e., smaller than the lower equivalence bound (Δ_*L*_ = -0.26) or larger than the upper equivalence bound (Δ_*U*_ = 0.26). If both tests were significant we concluded equivalence, implying that effects as extreme or more extreme than these values can be rejected. Results showed that the equivalence test based on Welch’s *t*-test was significant, *t*(283.73) = 1.74, *p* = .042, and therefore we reject the null hypothesis that the effect is smaller than -0.26 or larger than 0.26 (see Fig D in S2 Fig). Thus, if any intuitive cooperation effect is present, it is too small to be of interest. An intuitive cooperation effect only emerged if non-compliant participants were excluded (*p* < .001; see Fig 2 for regression plot).

## Study 2

In Study 2, we examined the influence of inducing trust on intuitive cooperation using a different cognitive process manipulation. Participants were exposed to a trust induction (high vs. low) before a one-shot public goods game in which they were instructed to decide intuitively or deliberatively. We expected that promoting high trust, compared to low trust, would increase cooperation to a greater degree when participants decided intuitively rather than deliberatively (hypothesis 1). We also anticipated greater cooperation when promoting intuition, compared to promoting deliberation (hypothesis 3). The study was pre-registered on AsPredicted (https://aspredicted.org/s6jh2.pdf).

### Methods

779 participants (229 men, 545 women, and 5 other, *M*_age_ = 37.21, *SD* = 11.65) participated in exchange for 30 pence and gained an additional 25 pence in the game. We applied the same exclusion criteria as Study 1. We conducted the study online to recruit a large sample and used Prolific (rather than MTurk) to target a population with little experience with similar studies, restricting participation to the United Kingdom.

Participants were exposed to a trust induction, played a one-shot public goods game, and were instructed to decide intuitively or deliberatively. The study had a 2 (trust induction: high vs. low) × 2 (cognitive process manipulation: intuition vs. deliberation) between-subjects design. The design was similar to Study 1. The main difference was that cognitive processes were manipulated through induction procedures rather than time constraints. In the cognitive process manipulation (presented on the decision screen), participants were instructed to rely on intuition or deliberation when making their decision in the public goods game. The manipulation was adapted from another induction that instructed participants to rely on emotion or reason [35]. Though the term “emotion” may be more familiar than “intuition” [35], relying on emotion could mean different things depending on the emotion that comes to mind, whereas relying on intuition more clearly indicates following one’s instinct. Our modified version was the following (changes to the intuition condition are presented in brackets):

Sometimes, people make decisions **deliberatively**, based on careful calculation. Other times, people make decisions **intuitively**, based on gut reactions.

[Sometimes, people make decisions **intuitively**, based on gut reactions.Other times, people make decisions **deliberatively**, based on careful calculation.]

Many people believe that deliberation [intuition] leads to good decision-making. When we use careful calculation [gut reactions], rather than gut reactions [careful calculation], we make satisfying decisions. Please make your transfer decision by **relying on deliberation**, rather than intuition [**relying on intuition**, rather than deliberation].

There were other differences from Study 1. First, we pre-registered the study to distinguish between confirmatory and exploratory analyses. Second, we increased the sample size to raise statistical power. To determine the sample size, we conducted an a priori power analysis in G*Power 3.1 to detect an interaction with a partial eta squared effect size of .02, with 95% power, given a 5% alpha level, and results showed that we needed 639 participants (and we aimed for 800 to increase power further) [36]. Third, we told participants that they were paired with other players (when in fact there was no pairing) to simplify the protocol. Participants were debriefed and paid the maximum amount they could have won in the game (25 pence). Fourth, we reduced the endowment in the game to 10 pence. Though stakes were much lower, online economic experiments using low stakes have found similar results to lab studies [37]. Fifth, the study was done in English rather than in Spanish. Finally, responses to the trust manipulation check were measured on a scale from 1 *not at all* to 7 *a great deal*, rather than from 0 *nothing* to 9 *very much*.

Participants completed additional measures. Understanding of the game was evaluated using two 6-option multiple choice questions. To check the influence of the cognitive process manipulation, we asked: “How did you make your decision?” from 1 *using only intuition* to 7 *using only deliberation* (reverse-scored); “To what extent did you rely on your gut reactions when making your decision?” from 1 *not at all* to 7 *a great deal*, and “To what extent did you rely on careful calculation when making your decision?” from 1 *not at all* to 7 *a great deal* (reverse-scored). We administered the trust manipulation check (and included questions of emotions), and measured experience with similar studies and demographics (age and gender), among other measures (see S1 Fig for summary of all tasks). We debriefed participants at the end of the study.

### Results

Prior to evaluating the hypotheses, we checked the impact of the manipulations, and examined understanding of the task and experience with similar studies. Though participants reported more trust in the high trust condition (*M* = 4.04, *SD* = 1.82, *n* = 391) than in the low trust condition (*M* = 3.83, *SD* = 1.83, *n* = 388), the difference was not significant, Welch’s *t*-test: *t*(776.91) = -1.59, *p* = .11, *M*_*diff*_ = -0.21, 95% CI [-0.47, 0.05]. However, the confidence interval indicated that the majority of plausible values for the effect were negative (i.e., high trust has a higher value than low trust). As predicted, participants reported making decisions significantly more intuitively (α = 0.75) in the intuition condition (*M* = 5.07, *SD* = 1.38, *n* = 382) than in the deliberation condition (*M* = 3.81, *SD* = 1.56, *n* = 397), Welch’s *t*-test: *t*(770.97) = -11.94, *p* < .001, *M*_*diff*_ = -1.26, 95% CI [-1.47, -1.05], indicating that the manipulation check succeeded. To rule out demand effects (i.e., participants responding based on perceived expectations without a truthful account of their behavior) we examined decision times (see Fig E in S2 Fig), and found significantly faster responses in the intuition condition (*M* = 17.39, *SD* = 11.25, *Mdn* = 15.35, *n* = 382) than in the deliberation condition (*M* = 21.27, *SD* = 14.52, *Mdn* = 18.91, *n* = 397), log_10_ decision times, Welch’s *t*-test: *t*(773.63) = 3.34, *p* < .001. Still, only 49% of participants answered both comprehension questions correctly and only 46% of participants had no experience with similar studies. Given the rates of experience and non-comprehension, we ran analyses excluding these participants, though these analyses may bias treatment estimates [30].

### Pre-registered analyses

#### Hypothesis 1

**Fig 3:**
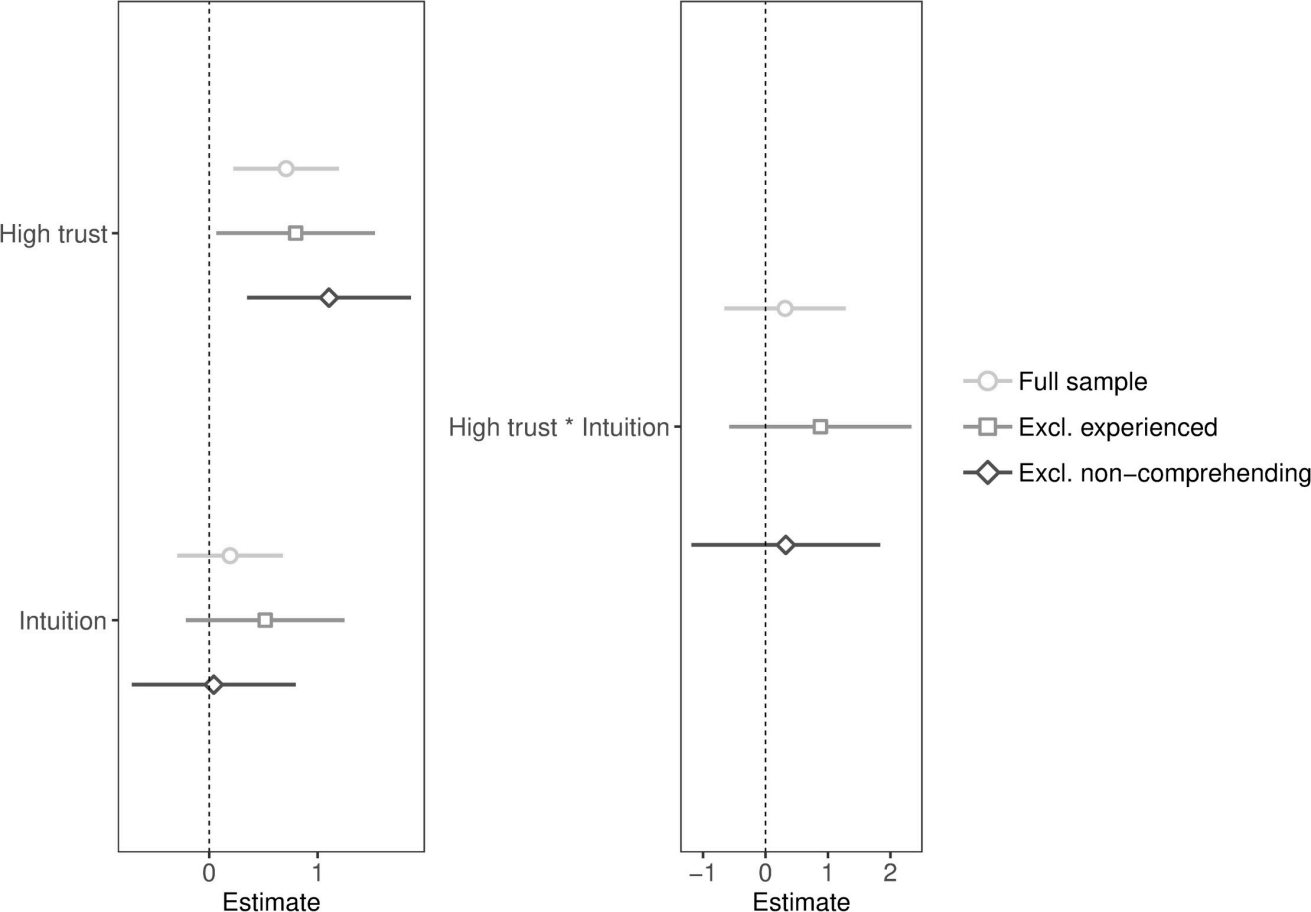
Unstandardized coefficients and 95% confidence intervals of public goods game contributions predicted by high trust and intuition (with and without interaction and exclusions). Coefficients of regression with main effects are presented on the left and coefficients of regression with interaction and simple effects are presented on the right (simple effects are not included in the plot).

We expected that promoting high trust, compared to low trust, would have a greater effect on cooperation when participants decided intuitively rather than deliberatively (see Table 2 for descriptive statistics and Fig 3 for regression plots). We first included main effects: high trust (1 = high trust, 0 = low trust) and intuition (1 = intuition, 0 = deliberation), and then added the interaction along with simple effects. Contrary to our hypothesis, we found no significant interaction between high trust and intuition (full sample: *p* = .53), even after exclusions (excluding experienced: *p* = .24; excluding non-comprehending: *p* = .67). We found a significant main effect of high trust (full sample: *p* = .004; excluding experienced: *p* = .033; excluding non-comprehending: *p* = .004), suggesting that the induction could have had the intended purpose, in the absence of awareness of changes in trust. We found no significant main effect of intuition (full sample: *p* = .44; excluding experienced: *p* = .17; excluding non-comprehending: *p* = .91). We deviated from the pre-registration given that we did not run regressions with controls (to maintain consistency with Study 1) or compared trust and emotions (we decided that this analysis was no longer meaningful). We also did not pre-register all the exclusion criteria but followed the criteria used in Study 1.

**Table 2:** Public goods game contributions, compliance, and comprehension depending on the condition.

Trust	Cognitive process	*n*	*M*_contribution_	*SD*_contribution_	*n*_naïve_	*n*_comprehend_
High	Intuition	191	7.87	3.15	92	89
	Deliberation	200	7.52	3.42	92	107
Low	Intuition	191	7.00	3.48	83	87
	Deliberation	197	6.96	3.73	93	95

Descriptive statistics of contributions are calculated in the full sample.

### Exploratory analyses

#### Hypothesis 3

**Fig 4:**
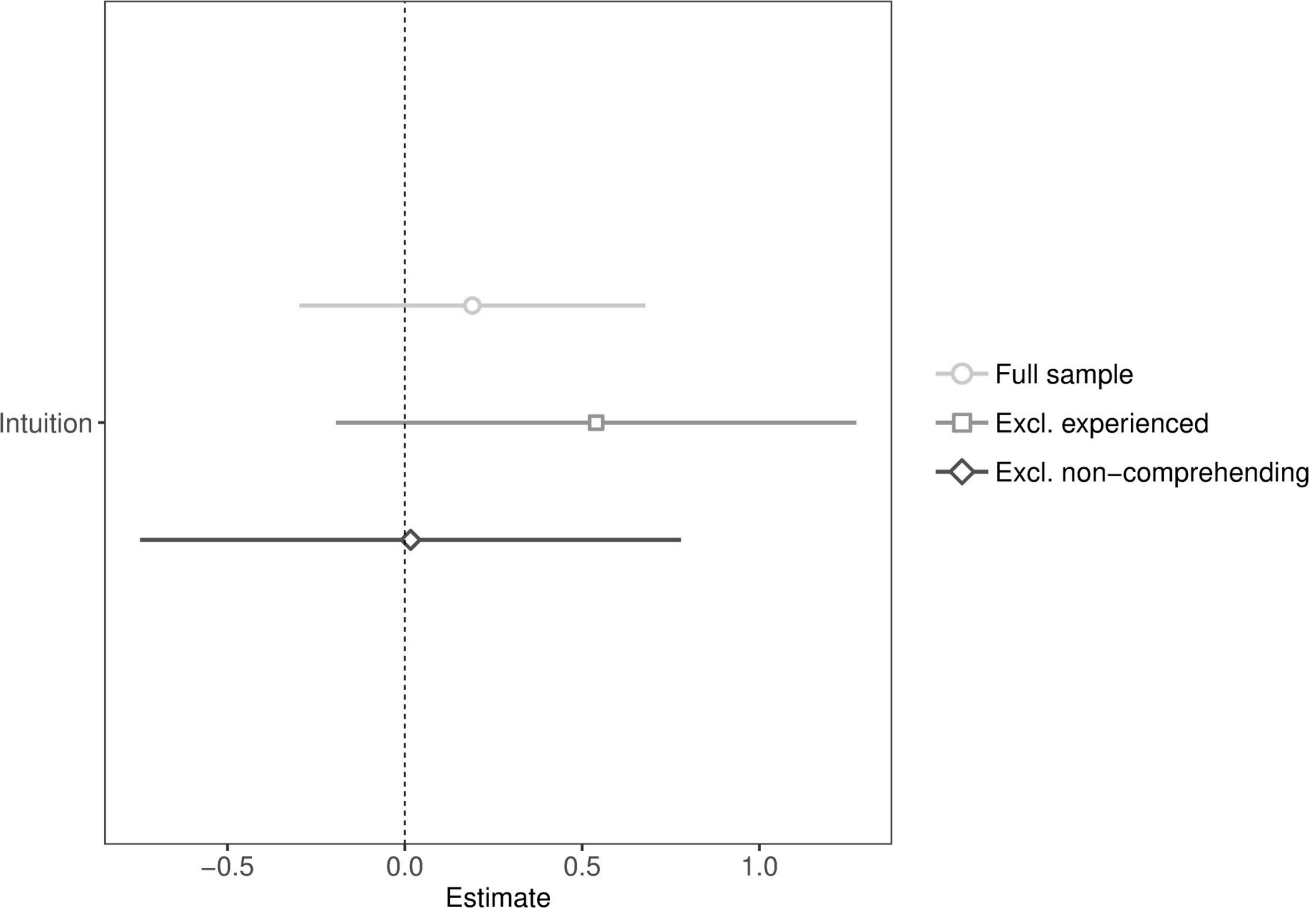
Unstandardized coefficients and 95% confidence intervals of public goods game contributions predicted by intuition (with and without exclusions).

We anticipated deciding intuitively to increase cooperation, compared to deciding deliberatively. Contrary to expectations, we found no significant effect of intuition (*p* = .44), even excluding experienced (*p* = .149) or non-comprehending participants (*p* = .97; see Fig 4 for regression plot), though the confidence interval of the sample excluding experienced participants indicated that the majority of plausible values for the effect were positive. We conducted equivalence tests (in the full sample) to check if the observed effect falls outside the equivalence bounds of -0.26 and 0.26 (Cohen’s *d*). We used the same smallest effect size of interest selected in Study 1 (see Study 1 for details on equivalence tests and justification of the smallest effect size of interest). Results showed that the equivalence test based on Welch’s *t*-test was significant, *t*(776.26) = -2.86, *p* = .002. Therefore, we reject the null hypothesis that the effect is smaller than -0.26 or larger than 0.26 (see Fig F in S2 Fig). Thus, if there is an effect, it is too small to be of interest.

## Discussion

In this paper, we examined the relation between trust and intuitive cooperation. Contrary to expectations, higher trust failed to promote intuitive cooperation, challenging correlations between trust and intuitive cooperation [7]. Higher trust increased cooperation regardless of the cognitive process. Also unexpectedly, preferring to process information intuitively or deliberatively failed to moderate the impact of trust on cooperation, contrary to research that found that reflective thinking moderates the effect of interactions on cooperation [19]. However, consistent with the SHH, people who prefer to process information intuitively cooperated more. Finally, intuition failed to increase cooperation compared to deliberation, and equivalence testing confirmed that this null result was due to the lack of an effect, rather than low statistical power. Though the author of an SHH meta-analysis argued that “future work may do well to focus on cognitive-processing manipulations other than time constraints (p. 13)” [11], we found no intuitive cooperation effect using an induction manipulation. Excluding non-comprehending or experienced participants did not alter the results considerably. An intuitive cooperation effect emerged if participants that did not comply with time constraints were excluded, but this result could be due to selection biases (e.g., artificially excluding less cooperative, slow responders from the time pressure condition) [12, 30]. Going beyond p-values and examining confidence intervals revealed considerable uncertainty regarding some conclusions [38]. Overall, most results failed to support the SHH (see Table 3 for unstandardized coefficients and *p* values).

**Table 3:** Unstandardized coefficients and *p* values for each hypothesis in each study.

Hypothesis	Test	Study	*p*	*b*	95% CI
(1) High trust, compared to low trust, would increase cooperation more when participants decide intuitively than deliberatively.	Interaction between high trust and time pressure.	1	.96	-35.16	[-1286.38, 1216.07]
	Interaction between high trust and intuition.	2	.53	0.31	[-0.66, 1.28]
(2a) High trust, compared to low trust, would have a greater effect on cooperation among participants that like an intuitive processing style.	Interaction between high trust and Faith in Intuition.	1	.31	-497.94	[-1469.12, 473.23]
(2b) High trust, compared to low trust, would have a greater effect on cooperation among participants that dislike a deliberative processing style.	Interaction between high trust and Need for Cognition.	1	.49	336.41	[-622.56, 1295.37]
(3) Intuition would increase cooperation, relative to deliberation.	Effect of time pressure.	1	.65	-147.14	[-774.47, 480.20]
	Effect of intuition.	2	.44	0.19	[-0.30, 0.68]

All results are calculated in the full sample. Contributions were measured from 0 to 8000 Colombian Pesos in Study 1, and from 0 to 10 pence in Study 2.

Given only limited support for the SHH, future work could clarify basic questions about the nature of intuition. An assumption in the SHH literature is that cognitive process manipulations, such as time constraints and induction procedures, influence intuition. However, as pointed out by critics of dual-process models, features of “intuition”, such as responding quickly or following instincts, may not be aligned, rendering it necessary to test this assumption [39]. In addition, more attention could be devoted to ruling out factors confounded with intuition. For example, promoting faster responses (i.e., time pressure) increases errors, perhaps by lowering the capacity to understand decisions or pay attention [14]. Moreover, the effect of deciding intuitively may depend on features of the game, such as familiarity with the task [16], discriminability of the choice options [40], and complexity of the strategies [41]. For instance, if the task is simple, deliberation may focus on introspection of motives, whereas if the task is complex, deliberation may focus on comparison of the choices. Future studies could examine how features of the game change the role of intuition and deliberation.

Our conclusions are qualified by several limitations. First, the trust induction had multiple weaknesses. The trust induction had a positive impact on cooperation in each study, but the manipulation check failed in Study 2. Though the trust induction could have had the intended effect without participants’ awareness of changes in trust, the result could also be explained by factors other than trust. Also, even when the trust manipulation was effective, the values were not particularly high or low in absolute terms. Future research could incorporate secondary manipulation checks (to provide convergent evidence), use stronger trust inductions (to push the means of the conditions apart), and include a control condition (to examine whether the effect, if found, is driven by high trust or low trust). Second, there was a low rate of compliance under time pressure (26%), which is close to the 34% found in the time constraint registered replication report [12]. Excluding non-compliant participants is not a viable solution given that it biases treatment estimates [12, 30]. Future studies could use procedures that reduce non-compliance, such as providing comprehension training [17], raising the time limit [17], familiarizing participants with the task [16], or incentivizing compliance [16]. Third, high rates of non-comprehension of the game may increase measurement error. Future studies could address this issue by conducting comprehension training [17], or assigning time limits to comprehension questions to correct for asymmetries in understanding [16]. Fourth, half of the Study 2 sample had experience with similar studies. Fifth, though we conducted analyses excluding participants based on posttreatment variables (compliance, comprehension, and experience), these results should be interpreted with caution given that treatment estimates may be biased [30]. Finally, though we tried to adequately justify our smallest effect size of interest, it was specified after results were known, and the interpretation of the equivalence tests hinges on our choice [31].

In sum, across two studies, we found that inducing trust failed to increase intuitive cooperation, processing information intuitively versus deliberatively failed to moderate the effect of trust, and there was little evidence that intuition promoted cooperation. Given the limited support for the SHH observed here, we suggest that future research should devote more attention to basic questions regarding the nature of intuition.

## Supporting information

S1 Appendix Study 3. (PDF)

S1 Table Regression tables. (PDF)

S1 Fig Tasks in each study. (PDF)

S2 Fig Histograms and plots. (PDF)
