# Correction: The role of trust in the social heuristics hypothesis

**DOI:** 10.1371/journal.pone.0241069

**Published:** 2021-01-27

**Authors:** Andres Montealegre, William Jimenez-Leal

After publication of this article [1], the authors contacted the journal office to correct two errors in the published article.

The reported number of participants in each session of Study 1 (8 or 12 participants) is incorrect. Most sessions were conducted in groups of either 8 or 12 participants. However, some sessions had more than 8 but fewer than 12 participants and some sessions had fewer than 8 participants. The correct number of participants in Study 1 sessions is accounted for in the corrected sentences below. The estimated frequency of participants per session in Study 1 is provided in S2 Table, which may be viewed below.

One pilot survey response from a previous version of the Study 2 survey is incorrectly included in the Study 2 dataset. The previous version of the survey had a different range for the dependent variable. As such, the incorrectly included response contains a value (16) for the dependent variable, which is outside of the possible range (0–10). The correct Study 2 methods and results which exclude the incorrectly included response are reported in the corrected sentences from Study 2 below. The authors confirm that excluding this response does not alter any of their conclusions (that is, all statistical tests that were significant remain significant and all statistical tests that were non-significant remain non-significant). Both a corrected dataset and R script are available from the Open Science Framework page listed in the Data Availability Statement, which remains unchanged.

The authors have provided updates to sentences in the Abstract, Study 1, and Study 2 sections to correct these errors. Please see the location of the error, the original text, and the author-corrected text here.

**Table 1:** 

Location	Original text	Corrected text
Abstract, eighth sentence	To evaluate these predictions, we conducted a lab study in Colombia and an online study in the United Kingdom (*N* = 1,066; one study was pre-registered).	To evaluate these predictions, we conducted a lab study in Colombia and an online study in the United Kingdom (*N* = 1,065; one study was pre-registered).
Study 1 section, Methods subsection, third paragraph, sixth sentence	The sessions were conducted in groups of 8 or 12 participants to guarantee that they could not identify others in their group.	Most sessions were conducted in groups of 8 or 12 participants. However, we ran some sessions with more than 8 but fewer than 12 participants and some sessions with fewer than 8 participants. The estimated frequency of participants per session is shown in S2 Table.
Study 2 section, Methods subsection, first sentence	779 participants (229 men, 545 women, and 5 other, *M*_age_ = 37.21, *SD* = 11.65) participated in exchange for 30 pence and gained an additional 25 pence in the game.	778 participants (228 men, 545 women, and 5 other, *M*_age_ = 37.23, *SD* = 11.65) participated in exchange for 30 pence and gained an additional 25 pence in the game.
Study 2 section, Results subsection, second sentence	Though participants reported more trust in the high trust condition (*M* = 4.04, *SD* = 1.82, *n* = 391) than in the low trust condition (*M* = 3.83, *SD* = 1.83, *n* = 388), the difference was not significant, Welch’s *t*-test: *t*(776.91) = -1.59, *p* = .11, *M*_*diff*_ = -0.21, 95% CI [-0.47, 0.05].	Though participants reported more trust in the high trust condition (*M* = 4.04, *SD* = 1.83, *n* = 390) than in the low trust condition (*M* = 3.83, *SD* = 1.83, *n* = 388), the difference was not significant, Welch’s *t*-test: *t*(775.96) = -1.59, *p* = .11, *M*_diff_ = -0.21, 95% CI [-0.47, 0.05].
Study 2 section, Results subsection, fourth sentence	As predicted, participants reported making decisions significantly more intuitively (α = 0.75) in the intuition condition (*M* = 5.07, *SD* = 1.38, *n* = 382) than in the deliberation condition (*M* = 3.81, *SD* = 1.56, *n* = 397), Welch’s *t*-test: *t*(770.97) = -11.94, *p* < .001, *M*_*diff*_ = -1.26, 95% CI [-1.47, -1.05], indicating that the manipulation check succeeded.	As predicted, participants reported making decisions significantly more intuitively (α = 0.75) in the intuition condition (*M* = 5.07, *SD* = 1.38, *n* = 382) than in the deliberation condition (*M* = 3.81, *SD* = 1.57, *n* = 396), Welch’s *t*-test: *t*(769.48) = -11.93, *p* < .001, *M*_diff_ = -1.26, 95% CI [-1.47, -1.05], indicating that the manipulation check succeeded.
Study 2 section, Results subsection, fifth sentence	To rule out demand effects (i.e., participants responding based on perceived expectations without a truthful account of their behavior) we examined decision times (see Fig E in S2 Fig), and found significantly faster responses in the intuition condition (*M* = 17.39, *SD* = 11.25, *Mdn* = 15.35, *n* = 382) than in the deliberation condition (*M* = 21.27, *SD* = 14.52, *Mdn* = 18.91, *n* = 397), log_10_ decision times, Welch’s *t*-test: *t*(773.63) = 3.34, *p* < .001.	To rule out demand effects (i.e., participants responding based on perceived expectations without a truthful account of their behavior) we examined decision times (see Fig E in S2 Fig), and found significantly faster responses in the intuition condition (*M* = 17.39, *SD* = 11.25, *Mdn* = 15.35, *n* = 382) than in the deliberation condition (*M* = 21.21, *SD* = 14.49, *Mdn* = 18.91, *n* = 396), log_10_ decision times, Welch’s *t*-test: *t*(772.46) = 3.29, *p* = .001.
Study 2 section, Pre-registered analyses subsection, Hypothesis 1 subheading, third sentence	Contrary to our hypothesis, we found no significant interaction between high trust and intuition (full sample: *p* = .53), even after exclusions (excluding experienced: *p* = .24; excluding non-comprehending: *p* = .67).	Contrary to our hypothesis, we found no significant interaction between high trust and intuition (full sample: *p* = .47), even after exclusions (excluding experienced: *p* = .24; excluding non-comprehending: *p* = .67).
Study 2 section, Pre-registered analyses subsection, Hypothesis 1 subheading, fourth sentence	We found a significant main effect of high trust (full sample: *p* = .004; excluding experienced: *p* = .033; excluding non-comprehending: *p* = .004), suggesting that the induction could have had the intended purpose, in the absence of awareness of changes in trust.	We found a significant main effect of high trust (full sample: *p* = .005; excluding experienced: *p* = .033; excluding non-comprehending: *p* = .004), suggesting that the induction could have had the intended purpose, in the absence of awareness of changes in trust.
Study 2 section, Pre-registered analyses subsection, Hypothesis 1 subheading, fifth sentence	We found no significant main effect of intuition (full sample: *p* = .44; excluding experienced: *p* = .17; excluding non-comprehending: *p* = .91).	We found no significant main effect of intuition (full sample: *p* = .39; excluding experienced: *p* = .17; excluding non-comprehending: *p* = .91).
Study 2 section, Exploratory analyses subsection, Hypothesis 3 subheading, second sentence	Contrary to expectations, we found no significant effect of intuition (*p* = .44), even excluding experienced (*p* = .149) or non-comprehending participants (*p* = .97; see Fig 4 for regression plot), though the confidence interval of the sample excluding experienced participants indicated that the majority of plausible values for the effect were positive.	Contrary to expectations, we found no significant effect of intuition (*p* = .39), even excluding experienced (*p* = .149) or non-comprehending participants (*p* = .97; see Fig 4 for regression plot), though the confidence interval of the sample excluding experienced participants indicated that the majority of plausible values for the effect were positive.
Study 2 section, Exploratory analyses subsection, Hypothesis 3 subheading, fifth sentence	Results showed that the equivalence test based on Welch’s *t*-test was significant, *t*(776.26) = -2.86, *p* = .002.	Results showed that the equivalence test based on Welch’s *t*-test was significant, *t*(775.40) = -2.78, *p* = .003.

Excluding the incorrectly included response changes the value of the High Deliberation row of Table 2. After removing the response, the mean contribution and standard deviation in the high trust—deliberation condition (*n* = 199) changes to 7.48 and 3.37, respectively. Please see the correct Table 2 here.

**Table 2:** Public goods game contributions, compliance, and comprehension depending on the condition.

Trust	Cognitive process	*n*	*M*_contribution_	*SD*_contribution_	*n*_naïve_	*n*_comprehend_
High	Intuition	191	7.87	3.15	92	89
	Deliberation	199	7.48	3.37	92	107
Low	Intuition	191	7.00	3.48	83	87
	Deliberation	197	6.96	3.73	93	95

Descriptive statistics of contributions are calculated in the full sample.

Updated versions of Figs 3 and 4 are not included since the changes are barely perceptually noticeable.

Excluding the incorrectly included response changes the value of the Study 2 rows of Table 3. After accounting for this error, the statistics of the interaction between high trust and intuition in Study 2 changes to *p* = .47, *b* = 0.36, 95% CI [-0.61, 1.32], and the statistics of effect of intuition in Study 2 changes to *p* = .39, *b* = 0.21, 95% CI [-0.27, 0.70]. Please see the correct Table 3 here.

**Table 3:** Unstandardized coefficients and *p* values for each hypothesis in each study.

Hypothesis	Test	Study	*p*	*b*	95% CI
(1) High trust, compared to low trust, would increase cooperation more when participants decide intuitively than deliberatively.	Interaction between high trust and time pressure.	1	.96	-35.16	[-1286.38, 1216.07]
	Interaction between high trust and intuition.	2	.47	0.36	[-0.61, 1.32]
(2a) High trust, compared to low trust, would have a greater effect on cooperation among participants that like an intuitive processing style.	Interaction between high trust and Faith in Intuition.	1	.31	-497.94	[-1469.12, 473.23]
(2b) High trust, compared to low trust, would have a greater effect on cooperation among participants that dislike a deliberative processing style.	Interaction between high trust and Need for Cognition.	1	.49	336.41	[-622.56, 1295.37]
(3) Intuition would increase cooperation, relative to deliberation.	Effect of time pressure.	1	.65	-147.14	[-774.47, 480.20]
	Effect of intuition.	2	.39	0.21	[-0.27, 0.70]

All results are calculated in the full sample. Contributions were measured from 0 to 8000 Colombian Pesos in Study 1, and from 0 to 10 pence in Study 2.

Excluding the incorrectly included response changes the value of the Table E and Table F sections of S1 Table. Please view the correct S1 Table below.

S2 Table is omitted from the list of Supporting Information. It can be viewed below.

Excluding the incorrectly included response changes the value of the means and standard deviations in S1 Fig. Please view the correct S1 Fig below.

Excluding the incorrectly included response changes the value of the Fig E and Fig F section of S2 Fig. Please view the correct S2 Fig below.

## Supporting information

S1 Table Regression tables. (DOCX)

S2 Table Estimated number of Study 1 participants per session. (DOCX)

S1 Fig Tasks in each study. (PDF)

S2 Fig Histograms and plots. (DOCX)
