# Supplementary material for: The role of trust in the social heuristics hypothesis
Source: PLoS One. 2019 May 10;14(5):e0216329. doi: 10.1371/journal.pone.0216329 (PMC6510443; doi:10.1371/journal.pone.0216329)
Supplement: S1 Appendix — (PDF) [file pone.0216329.s001.pdf]

S1 Appendix. Study 3.

Study 3

In Study 3, we assessed whether self-report or behavioral trust measures were more predictive of intuitive cooperation. Participants played a one-shot anonymous public goods game under different time constraints, and we measured trust using the trust game [1, 2], the Propensity to Trust Survey [3], and the World Value Survey trust question. We expected higher scores on the trust measures to be associated with more contributions under time pressure than under time delay, though we had no expectation regarding which measure would a better predictor. We also tested whether contributions were greater under time pressure than under time delay.

Methods

113 participants participated in this study in exchange for $8,000 Colombian Pesos and gained an additional amount in the public goods game and the trust game. We aimed for a sample size of 150 participants, but ended up with less due to resource and time constraints. We applied the same exclusion criteria as in previous studies and, additionally, excluded 13 participants that participated in Study 1. Our main analyses were restricted to player one in the trust game and, thus, we ended up with a sample of 57 participants (32 men, 24 women, and 1 other, $M_{\text{age}} = 21.91$, $SD = 2.89$). Note that the smaller sample (compared to the expected sample) reduced our power considerably and therefore our findings should be replicated with larger samples before drawing any strong conclusions. We conducted this study in the lab (at a public university in Colombia) to target a population with little experience with similar studies. The study was conducted in Spanish.

In the study, participants played a public goods game under different time constraint manipulations (time pressure or time delay), participated in a trust game (behavioral trust measure), and answered the Propensity to Trust Survey and the World Value Survey trust question (self-report trust measures). The public goods game implementation was identical to Study 1, except that participants were given a $4,000 Colombian Pesos endowment and there was no random payment. Participants were exposed to all trust measures, but were assigned to be either player one or two in the trust game to avoid deception (though we restricted main analyses to player one). We randomized the order of the games (public goods game and trust game) to examine order effects [4]. After the games, participants answered the Propensity to Trust Survey [3], the World Value Survey trust question, and other questions: experience with economic games and research participation more generally [5], social capital [6], Perceived Awareness of the Research Hypothesis scale and other questions about demand effects [5, 7], demographics (including education given that we did not recruit only undergraduates), how many of the people in the room participants knew, and whether they participated in Study 1. We now explain the trust measures.

The trust game is a behavioral measure of trust [1, 2]. In the game, there are two players in different rooms and the game has two stages. In the first stage, player one must decide how much of a show-up fee to send to player two (both players know that the money that player one sends to player two will be tripled), in the second stage, player two receives the tripled amount and decides how much of his or her money to keep (which in our setup is the amount sent by player one tripled plus the show-up fee) and how much to return to player one. Given space limitations, we ran separate sessions with players one and two and paid participants at the end of the week. The game involved real monetary incentives, with each player assigned $4,000 Colombian Pesos as a show-up fee. Trust was measured as the

amount sent by player one to player two. Participants had to type the amount they wanted to send/return in a box. The trust game was anonymous and participants were not able to see the screens of other participants. The game was done on a computer, but players two were informed about the amount received in a separate sheet. We avoided random payment schemes, given that a trust game meta-analysis showed that they reduce the amount sent by player one [2]. We included four comprehension questions (text entered) and asked them why they made their decision (an open-ended question).

The Propensity to Trust Survey is a validated 21-item self-report instrument composed of two scales that measure individual differences in trust and trustworthiness. Research has demonstrated that the trust and trustworthiness scales are reliable, related to several of the Big Five personality traits, and predict the behavior of people in a trust game better than the Big Five scales [3]. We administered the trust scale (7 items), included 14 Big Five filler items [8] to conceal the purpose of the questionnaire, and randomized the order of the questions. Trust was measured by computing an average with the items of the trust scale after reverse scoring them and responses were measured on a 6-point scale from 1 *very inaccurate* to 6 *very accurate*.

The World Value Survey trust question was the one used in previous research on intuitive cooperation [9]. The question asked respondents "To what extent do you feel you can trust other people that you interact with in your daily life?" and responses were measured on a 10-point scale ranging from 1 *very little* to 10 *very much*. We also included five interpersonal trust questions for exploratory purposes, 13 filler items to conceal the purpose of the questionnaire, and randomized the order of the questions. The questions were taken from the World Value Survey.

Results

We examined whether the cognitive process manipulation check succeeded and evaluated understanding of the games in the relevant sub-sample of trust game players one. We found that participants responded faster under time pressure (M = 12.65, SD = 5.82, Mdn = 11.66, n = 29) than under time delay (M = 27.14, SD = 13.68, Mdn = 24.69, n = 28). Decision times were right-skewed and we therefore applied a $\log_{10}$ transformation (see Fig 1). Though the difference between $\log_{10}$ decision times in the conditions was significant, $t(50.66) = 4.95$, $p < .001$, the median decision time under time pressure was higher than 10 seconds and only 31% of participants complied under time pressure, compared to 89% under time delay. Concerning comprehension of the games, only 61% of participants passed both checks in the public goods game and only 39% passed the four checks in the trust game. Overall, participants answered faster under time pressure (compared to time delay), but there was a high rate of non-compliance under time pressure and only a subset of participants fully understood each game (and the problem was greater in the trust game perhaps due to a more stringent comprehension check).

Fig 1. Distribution of decision times (left) and distribution of log10 decision times (right).

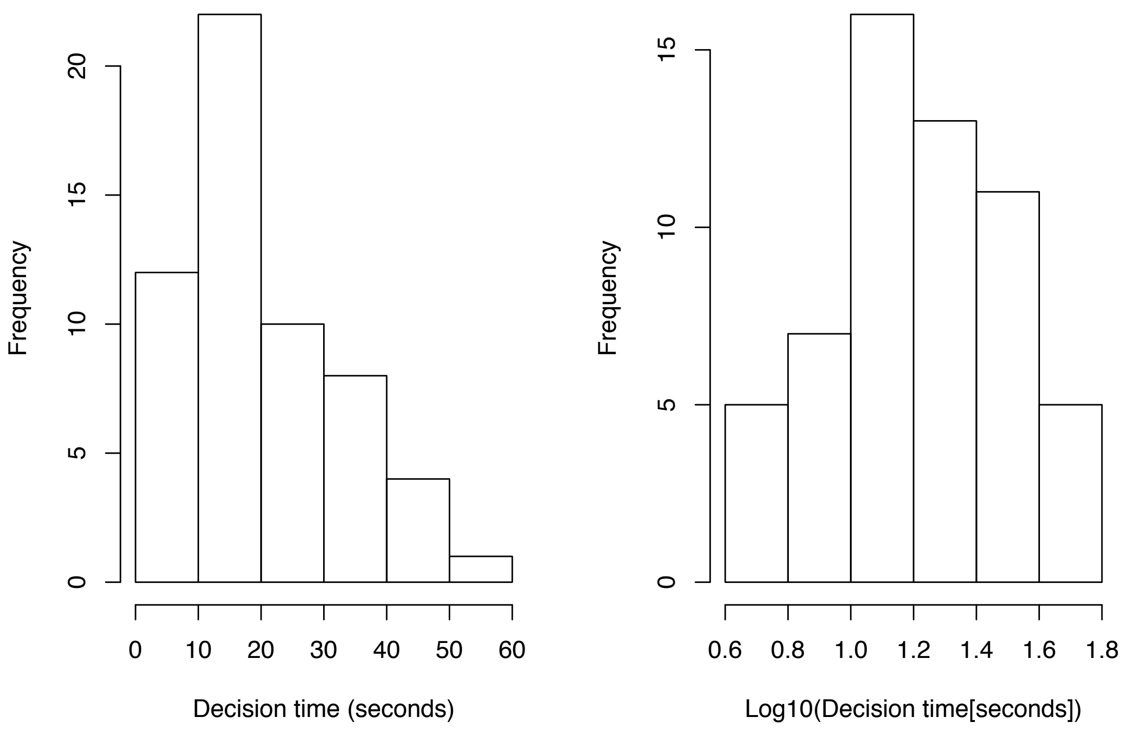

We used dominance analysis to compare the relative importance of the trust measures in predicting intuitive cooperation [10, 11]. In the trust game, trust was measured as the amount sent from player one to player two (M = 2,612.35, SD = 1,280.77, n = 57); in the Propensity to Trust Survey, trust was measured using the trust scale (M = 3.63, SD = 0.88, n = 57; α = 0.74); and in the World Value Survey, trust was measured using a survey question (M = 7.39, SD = 1.61, n = 57). When predictor variables are orthogonal, determining the relative importance of those predictors is easy and straightforward (e.g., using squared bivariate correlations between each of the predictors and the criterion), but when predictor variables are correlated, estimation of relative importance is still possible, but it becomes more complex [12, 13]. When predictors are correlated, the preferred

statistical tools are dominance analysis [11] or relative weight analysis [12]. It has been shown that these two approaches yield virtually identical estimates of relative weights [12]. Specifically, dominance analysis estimates the importance of a predictor in multiple regression by evaluating its contribution to the R^2 across all possible subset regressions. There are different forms of dominance ranging from complete dominance (strongest) to general dominance (weakest; note that if dominance is achieved in a stronger form, then it is also achieved in the weaker forms): 1) complete: additional contribution of one predictor over another needs to be satisfied in every subset model; 2) conditional: average additional contribution needs to be satisfied across all models of a fixed size, and 3) general: average additional contribution needs to be satisfied across all models [11]. We used *residualized* dominance analysis to test the predictive power of interactions, given that the standard version does not provide "clean" estimates of higher order effects [13]. Specifically, we compared the predictive power of the interactions between the trust measures and time pressure in predicting contributions. Dominance analysis was conducted using the yhat R package [14]. Results showed that the World Value Survey × time pressure interaction completely dominated the trust game × time pressure and the Propensity to Trust Survey × time pressure interactions. Though dominance could not be established between the Propensity to Trust Survey × time pressure and the trust game × time pressure interactions at the complete level, the Propensity to Trust Survey × time pressure interaction conditionally dominated the trust game × time pressure interaction. While the World Value Survey trust question seems to be the better general predictor of intuitive cooperation, followed by the Propensity to Trust Survey, and the trust game, the average contribution of each predictor to the R^2 is quite small.

We selected the interaction that was the better predictor (World Value Survey ×

time pressure) and tested its significance (see Table 1 for regressions). In the regressions, we first entered main effects: World Value Survey and time pressure (1 = time pressure, 0 = time delay), then added the interaction with its components, and finally included controls: age, gender (1 = female, 0 = male), socioeconomic status (from 1 to 6), knows other participants (1 = yes, 0 = no), and order of the games (1 = TG then PGG, 0 = PGG then TG). We examined main effects and found a significant positive effect of World Value Survey (column 1, $p = .003$) but not of time pressure (column 1, $p = .32$), and found that the World Value Survey × time pressure interaction was not significant (column 2, $p = .39$).

Table 1. Multiple linear regressions with public goods game contributions predicted by the World Value Survey and time pressure (with and without interaction and controls).

	Dependent variable:		
	PGG contribution		
	(1)	(2)	(3)
World Value Survey (WVS)	350.605***	449.794***	559.466***
	(112.906)	(160.808)	(163.373)
Time pressure (TP)	-360.909	1,088.434	987.903
	(360.845)	(1,708.135)	(1,749.669)
TP × WVS		-196.503	-233.079
		(226.339)	(231.275)
Age			65.749
			(59.064)
Gender			-542.287
			(352.790)
Socioeconomic status			590.776**
			(224.423)
Knows other participants			-640.109*
			(364.345)
Order of the games			353.997
			(354.290)
Constant	-39.201	-733.523	-4,268.049**
	(828.948)	(1,153.212)	(1,917.120)
Observations	57	57	56
R^2	0.152	0.164	0.354
Adjusted R^2	0.121	0.117	0.244
Residual Std. Error	1,323.027 (df = 54)	1,326.055 (df = 53)	1,223.015 (df = 47)
F Statistic	4.858** (df = 2; 54)	3.475** (df = 3; 53)	3.218*** (df = 8; 47)

Note: *p<0.1; **p<0.05; ***p<0.01
Standard errors are reported in parentheses

We evaluated whether there was an effect of time pressure on contributions using the full sample ($N = 113$). Though the manipulation check on the $\log_{10}$ decision times succeeded, $t(104.66) = 6.66$, $p < .001$, we found no significant differences in contributions between time pressure ($M = 2{,}345.82$, $SD = 1{,}198.44$, $n = 57$) and time delay ($M = 2{,}448.48$, $SD = 1{,}357.18$, $n = 56$), $t(108.82) = 0.43$, $p = .67$, suggesting that time constraints did not influence contributions. We used equivalence tests to examine whether the effect falls outside the equivalence bounds of -0.26 and 0.26 (Cohen's *d*; see Study 1 for details on equivalence tests and the justification of the bounds). Whereas the *t*-test against the lower value suggests that we cannot reject effects as extreme or more extreme than -0.26, the *t*-test on the upper value indicates we can reject effects as extreme or more extreme than 0.26. The equivalence test based on Welch's *t*-test is therefore not significant, $t(108.82) = 0.96$, $p = .17$, implying that we cannot reject the null hypothesis that the effect is smaller than -0.26 or larger than 0.26 (see Fig 2).

Fig 2. Equivalence test for effect of time pressure on public goods game contributions.

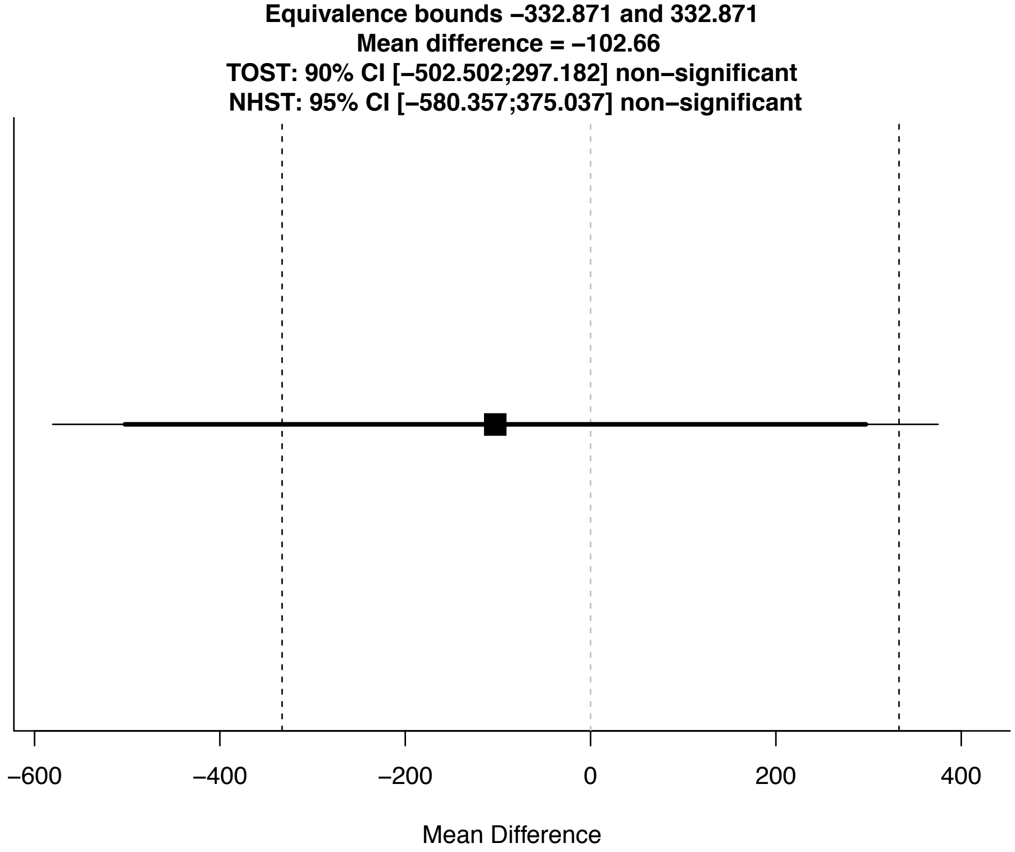

References

1. Berg J, Dickhaut J, McCabe K. Trust, Reciprocity, and Social History. Games and Economic Behavior. 1995;10(1):122-42. doi: http://dx.doi.org/10.1006/game.1995.1027.
2. Johnson ND, Mislin AA. Trust games: A meta-analysis. Journal of Economic Psychology. 2011;32(5):865-89. doi: http://dx.doi.org/10.1016/j.joep.2011.05.007.
3. Evans AM, Revelle W. Survey and behavioral measurements of interpersonal trust. Journal of Research in Personality. 2008;42(6):1585-93. doi: http://dx.doi.org/10.1016/j.jrp.2008.07.011.
4. Brañas P, Barreda In. Experimentos en economía. In: Brañas P, editor. Economía experimental y del comportamiento: Antoni Bosch editor; 2011.
5. Bouwmeester S, Verkoeijen PPJL, Aczel B, Barbosa F, Bègue L, Brañas-Garza P, et al. Registered Replication Report: Rand, Greene, and Nowak (2012). Perspectives on Psychological Science. 2017;12(3):527-42. doi: 10.1177/1745691617693624. PubMed PMID: 28475467.
6. Cárdenas JC, Chong A, Ñopo H. Stated social behavior and revealed actions: Evidence from six Latin American countries. Journal of Development Economics. 2013;104:16-33. doi: http://dx.doi.org/10.1016/j.jdeveco.2013.04.002.

7. Rubin M. The Perceived Awareness of the Research Hypothesis Scale: Assessing the influence of demand characteristics. 2017.
8. John OP, Naumann LP, Soto CJ. Paradigm shift to the integrative Big Five trait taxonomy: History, measurement, and conceptual issues. Handbook of personality: Theory and research, 3rd ed. New York, NY, US: Guilford Press; 2008. p. 114-58.
9. Rand DG, Greene JD, Nowak MA. Spontaneous giving and calculated greed. Nature. 2012;489(7416):427-30. doi: http://www.nature.com/nature/journal/v489/n7416/abs/nature11467.html - supplementary-information.
10. Budescu DV. Dominance analysis: A new approach to the problem of relative importance of predictors in multiple regression. Psychological Bulletin. 1993;114(3):542-51. doi: 10.1037/0033-2909.114.3.542.
11. Azen R, Budescu DV. The dominance analysis approach for comparing predictors in multiple regression. Psychological Methods. 2003;8(2):129-48. doi: 10.1037/1082-989X.8.2.129.
12. Tonidandel S, LeBreton JM. Relative Importance Analysis: A Useful Supplement to Regression Analysis. Journal of Business and Psychology. 2011;26(1):1-9.
13. LeBreton JM, Tonidandel S, Krasikova DV. Residualized Relative Importance Analysis:A Technique for the Comprehensive Decomposition of Variance in Higher Order Regression Models. Organizational Research Methods. 2013;16(3):449-73. doi: 10.1177/1094428113481065.
14. Nimon K, Oswald F, Roberts J. yhat: Interpreting Regression Effects. R package version 2.0-0. 2013.
