# Supplementary material for: The role of trust in the social heuristics hypothesis
Source: PLoS One. 2019 May 10;14(5):e0216329. doi: 10.1371/journal.pone.0216329 (PMC6510443; doi:10.1371/journal.pone.0216329)
Supplement: S1 Table — (PDF) [file pone.0216329.s002.pdf]

S1 Table. Regression tables.

Table A. Public goods game contributions predicted by high trust and time pressure with and without interaction and exclusions (Study 1).

	Dependent variable:					
	PGG contribution					
	Full sample		Excluding non-compliant		Excluding non-comprehending	
	(1)	(2)	(3)	(4)	(5)	(6)
High trust (HT)	652.257**	670.037	466.745	569.895	-1.451	769.236
	(317.252)	(452.047)	(417.907)	(472.467)	(433.499)	(626.397)
Time pressure (TP)	-117.516	-99.737	1,798.635***	2,086.730***	28.118	791.842
	(317.252)	(452.047)	(499.873)	(789.595)	(433.700)	(623.561)
HT × TP		-35.157		-482.342		-1,463.296*
		(635.662)		(1,021.678)		(863.131)
Constant	4,824.047***	4,814.657***	4,664.794***	4,612.413***	5,084.277***	4,633.875***
	(280.754)	(328.526)	(317.114)	(336.684)	(400.763)	(478.863)
Observations	287	287	165	165	161	161
R^2	0.015	0.015	0.085	0.087	0.00003	0.018
Adjusted R^2	0.008	0.005	0.074	0.069	-0.013	-0.001
Residual Std. Error	2,684.376 (df = 284)	2,689.100 (df = 283)	2,665.933 (df = 162)	2,672.350 (df = 161)	2,724.874 (df = 158)	2,708.856 (df = 157)
F Statistic	2.221 (df = 2; 284)	1.477 (df = 3; 283)	7.548*** (df = 2; 162)	5.082*** (df = 3; 161)	0.002 (df = 2; 158)	0.960 (df = 3; 157)

Note: *p<0.1; **p<0.05; ***p<0.01
Standard errors are reported in parentheses

Table B. Public goods game contributions predicted by high trust, time pressure, and Faith in Intuition with and without interactions and exclusions (Study 1).

	Dependent variable:					
	PGG contribution					
	Full sample		Excluding non-compliant		Excluding non-comprehending	
	(1)	(2)	(3)	(4)	(5)	(6)
High trust (HT)	607.813*	2,309.765	438.281	1,941.931	-31.950	3,065.703
	(314.811)	(1,724.309)	(414.772)	(2,117.860)	(430.612)	(2,548.743)
Time pressure (TP)	-130.849	1,386.840	1,795.286***	5,372.518**	-13.549	2,335.621
	(314.360)	(1,708.237)	(495.809)	(2,583.190)	(431.089)	(2,554.198)
Faith in Intuition (FI)	614.036**	1,049.857**	582.159*	1,000.582**	653.740*	1,236.527*
	(244.025)	(416.460)	(303.918)	(462.394)	(356.372)	(678.101)
HT × TP		-17.214		-427.875		-1,344.068
		(631.421)		(1,013.444)		(862.546)
HT × FI		-497.943		-423.874		-700.791
		(493.365)		(615.412)		(732.021)
TP × FI		-443.868		-988.731		-485.080
		(492.168)		(732.968)		(731.638)
Constant	2,774.502***	1,298.418	2,725.376**	1,286.669	2,882.779**	499.239
	(860.699)	(1,432.345)	(1,060.209)	(1,572.756)	(1,264.307)	(2,317.005)
Observations	287	287	165	165	161	161
R^2	0.037	0.043	0.106	0.118	0.021	0.045
Adjusted R^2	0.027	0.022	0.089	0.085	0.002	0.008
Residual Std. Error	2,659.528 (df = 283)	2,665.713 (df = 280)	2,644.238 (df = 161)	2,650.095 (df = 158)	2,704.706 (df = 157)	2,697.605 (df = 154)
F Statistic	3.619** (df = 3; 283)	2.083* (df = 6; 280)	6.338*** (df = 3; 161)	3.536*** (df = 6; 158)	1.123 (df = 3; 157)	1.202 (df = 6; 154)
Note:						*p<0.1; **p<0.05; ***p<0.01 Standard errors are reported in parentheses

Table C. Public goods game contributions predicted by high trust, time pressure, and Need for Cognition with and without interactions and exclusions (Study 1).

	Dependent variable:					
	PGG contribution					
	Full sample		Excluding non-compliant		Excluding non-comprehending	
	(1)	(2)	(3)	(4)	(5)	(6)
High trust (HT)	669.843**	-655.854	466.152	-132.746	7.566	-1,900.215
	(316.983)	(1,957.657)	(419.038)	(2,400.053)	(434.430)	(2,587.027)
Time pressure (TP)	-90.988	-1,441.011	1,793.403***	-314.242	23.230	-4,794.978*
	(317.305)	(2,001.048)	(501.435)	(3,542.212)	(434.491)	(2,627.873)
Need for Cognition (NFC)	335.347	51.091	107.175	-66.037	226.476	-817.020
	(241.070)	(377.756)	(298.647)	(416.513)	(330.943)	(565.190)
HT × TP		89.908		-419.870		-1,350.140
		(640.211)		(1,034.097)		(862.906)
HT × NFC		336.406		181.606		721.079
		(487.163)		(608.397)		(664.844)
TP × NFC		342.989		604.735		1,469.761**
		(500.185)		(869.255)		(669.907)
Constant	3,504.072***	4,616.392***	4,250.965***	4,866.288***	4,220.163***	7,672.169***
	(989.420)	(1,502.295)	(1,196.189)	(1,636.769)	(1,324.982)	(2,154.783)
Observations	287	287	165	165	161	161
R^2	0.022	0.026	0.086	0.091	0.003	0.053
Adjusted R^2	0.012	0.005	0.069	0.056	-0.016	0.016
Residual Std. Error	2,679.968 (df = 283)	2,689.580 (df = 280)	2,673.130 (df = 161)	2,691.339 (df = 158)	2,729.470 (df = 157)	2,686.426 (df = 154)
F Statistic	2.131* (df = 3; 283)	1.221 (df = 6; 280)	5.048*** (df = 3; 161)	2.628** (df = 6; 158)	0.158 (df = 3; 157)	1.427 (df = 6; 154)
Note:						*p<0.1; **p<0.05; ***p<0.01<br>Standard errors are reported in parentheses

Table D. Public goods game contributions predicted by time pressure with and without exclusions (Study 1).

	Dependent variable:		
	PGG contribution		
	Full sample	Excluding non-compliant	Excluding non-comprehending
	(1)	(2)	(3)
Time pressure	-147.136	1,851.755***	28.310
	(318.714)	(497.983)	(428.553)
Constant	5,168.549***	4,901.812***	5,083.429***
	(226.540)	(235.816)	(309.550)
Observations	287	165	161
R^2	0.001	0.078	0.00003
Adjusted R^2	-0.003	0.073	-0.006
Residual Std. Error	2,699.531 (df = 285)	2,667.955 (df = 163)	2,716.292 (df = 159)
F Statistic	0.213 (df = 1; 285)	13.827*** (df = 1; 163)	0.004 (df = 1; 159)

Note: *p<0.1; **p<0.05; ***p<0.01
Standard errors are reported in parentheses

Table E. Public goods game contributions predicted by high trust and intuition with and without interaction and exclusions (Study 2).

	Dependent variable:					
	PGG contribution					
	Full sample		Excluding experienced		Excluding non-comprehending	
	(1)	(2)	(3)	(4)	(5)	(6)
High trust (HT)	0.709***	0.556	0.797**	0.370	1.104***	0.952*
	(0.247)	(0.346)	(0.371)	(0.517)	(0.383)	(0.526)
Intuition (INT)	0.193	0.036	0.517	0.067	0.043	-0.126
	(0.247)	(0.350)	(0.371)	(0.531)	(0.384)	(0.553)
HT × INT		0.314		0.879		0.326
		(0.495)		(0.742)		(0.770)
Constant	6.887***	6.964***	6.842***	7.054***	6.677***	6.758***
	(0.213)	(0.246)	(0.318)	(0.365)	(0.332)	(0.383)
Observations	779	779	360	360	378	378
R^2	0.011	0.012	0.018	0.022	0.022	0.022
Adjusted R^2	0.009	0.008	0.013	0.014	0.016	0.014
Residual Std. Error	3.449 (df = 776)	3.451 (df = 775)	3.520 (df = 357)	3.518 (df = 356)	3.724 (df = 375)	3.728 (df = 374)
F Statistic	4.413** (df = 2; 776)	3.074** (df = 3; 775)	3.359** (df = 2; 357)	2.709** (df = 3; 356)	4.146** (df = 2; 375)	2.818** (df = 3; 374)

Note: *p<0.1; **p<0.05; ***p<0.01
Standard errors are reported in parentheses

Table F. Public goods game contributions predicted by intuition with and without exclusions (Study 2).

	Dependent variable:		
	PGG contribution		
	Full sample	Excluding experienced	Excluding non-comprehending
	(1)	(2)	(3)
Intuition	0.190	0.539	0.016
	(0.248)	(0.373)	(0.388)
Constant	7.244***	7.238***	7.262***
	(0.174)	(0.260)	(0.265)
Observations	779	360	378
R^2	0.001	0.006	0.00000
Adjusted R^2	-0.001	0.003	-0.003
Residual Std. Error	3.465 (df = 777)	3.537 (df = 358)	3.760 (df = 376)
F Statistic	0.587 (df = 1; 777)	2.090 (df = 1; 358)	0.002 (df = 1; 376)

Note: *p<0.1; **p<0.05; ***p<0.01
Standard errors are reported in parentheses
