# Supplementary material for: The role of trust in the social heuristics hypothesis
Source: PLoS One. 2019 May 10;14(5):e0216329. doi: 10.1371/journal.pone.0216329 (PMC6510443; doi:10.1371/journal.pone.0216329)
Supplement: S1 Fig — (PDF) [file pone.0216329.s003.pdf]

Study 1

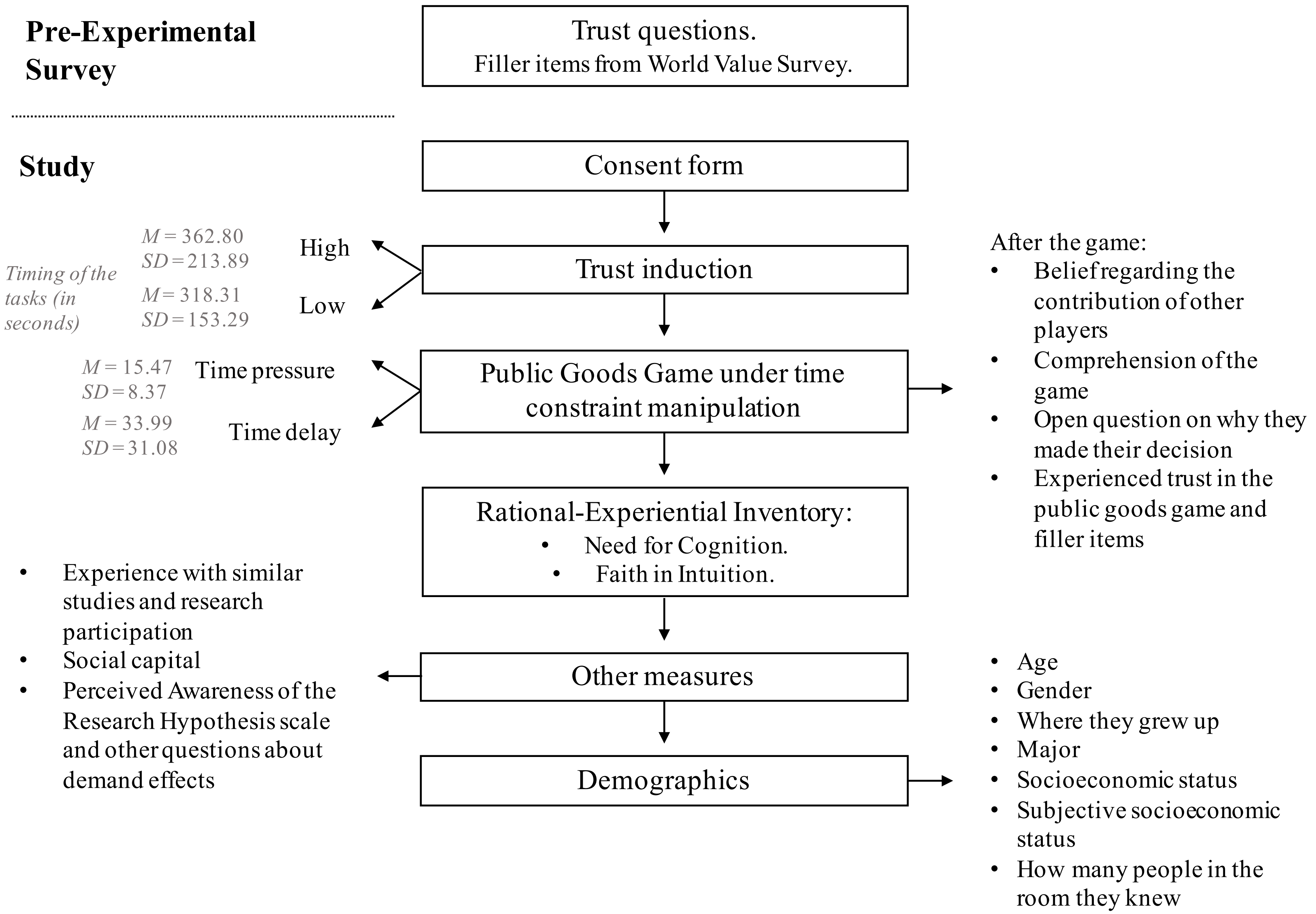
Pre-Experimental Survey
Trust questions.
Filler items from World Value Survey.
Study
Consent form
Timing of the tasks (in seconds)
M = 362.80
SD = 213.89
High
M = 318.31
SD = 153.29
Low
Trust induction
M = 15.47
SD = 8.37
Time pressure
M = 33.99
SD = 31.08
Time delay
Public Goods Game under time constraint manipulation
After the game:
• Belief regarding the contribution of other players
• Comprehension of the game
• Open question on why they made their decision
• Experienced trust in the public goods game and filler items
Rational-Experiential Inventory:
• Need for Cognition.
• Faith in Intuition.
• Experience with similar studies and research participation
• Social capital
• Perceived Awareness of the Research Hypothesis scale and other questions about demand effects
Other measures
Demographics
• Age
• Gender
• Where they grew up
• Major
• Socioeconomic status
• Subjective socioeconomic status
• How many people in the room they knew

Study 2

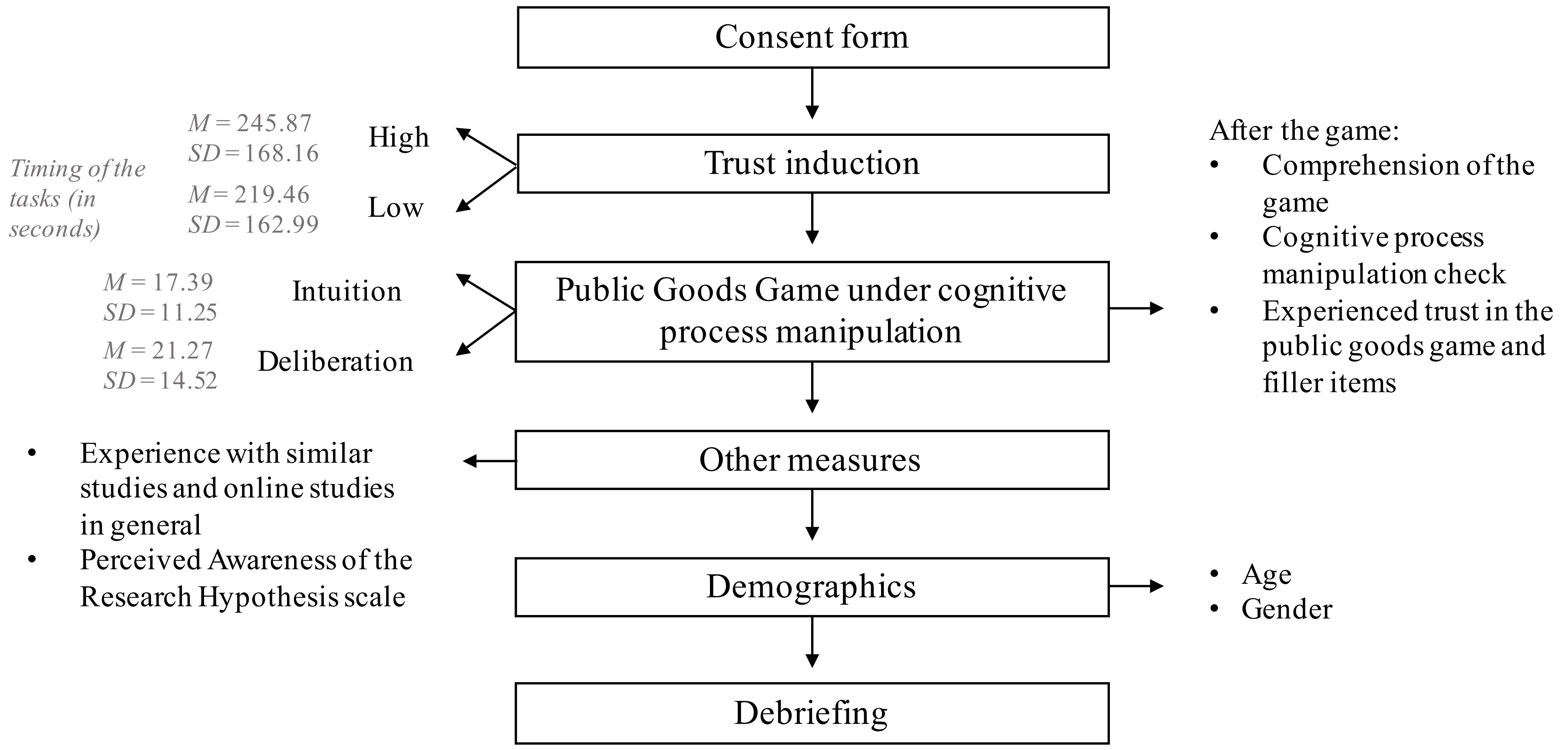
Consent form
Trust induction
High
Low
Timing of the tasks (in seconds)
M = 245.87
SD = 168.16
M = 219.46
SD = 162.99
Public Goods Game under cognitive process manipulation
Intuition
Deliberation
M = 17.39
SD = 11.25
M = 21.27
SD = 14.52
After the game:
• Comprehension of the game
• Cognitive process manipulation check
• Experienced trust in the public goods game and filler items
Other measures
• Experience with similar studies and online studies in general
• Perceived Awareness of the Research Hypothesis scale
Demographics
• Age
• Gender
Debriefing

Study 3

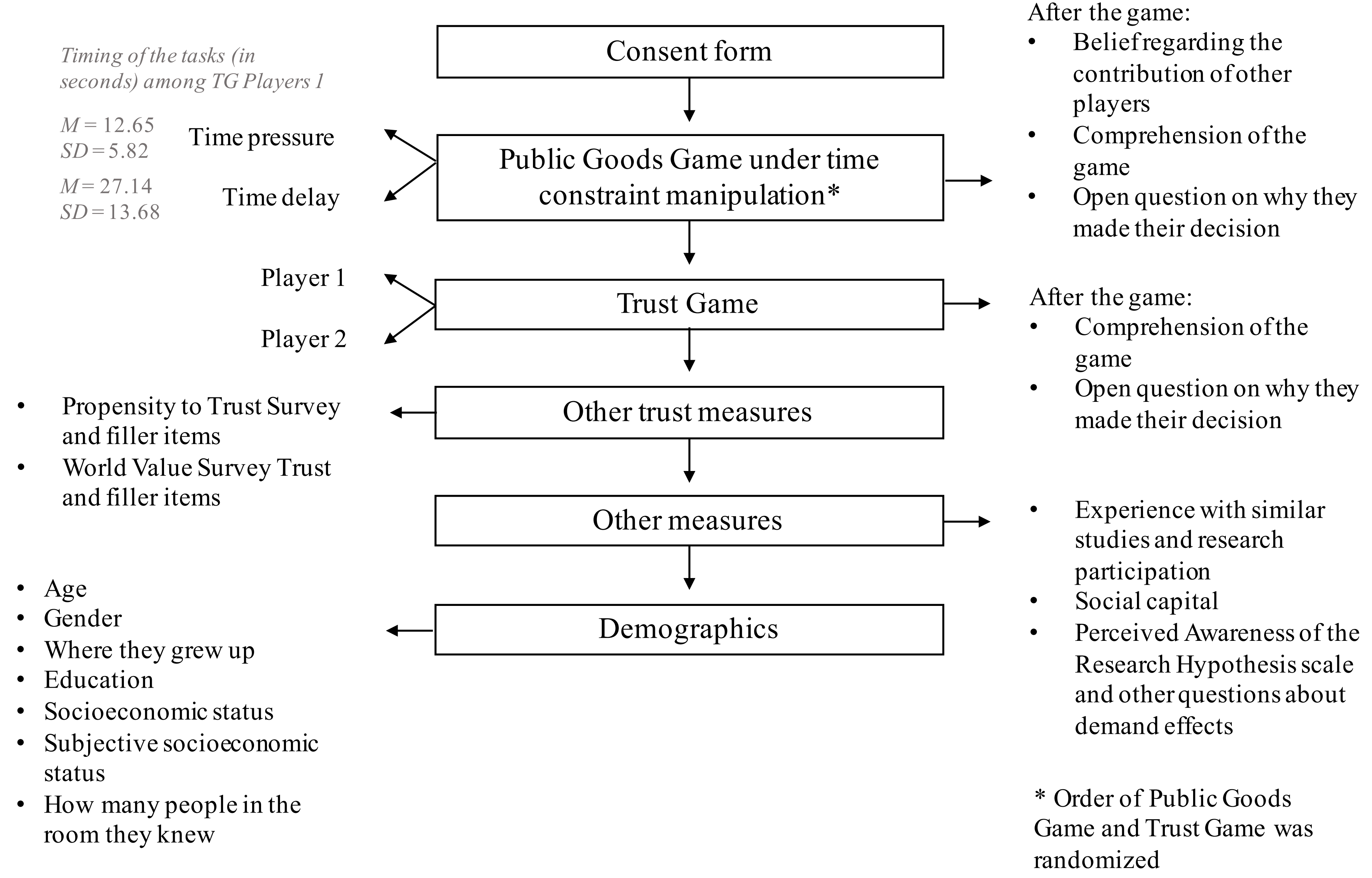
Timing of the tasks (in seconds) among TG Players 1
M = 12.65
SD = 5.82
M = 27.14
SD = 13.68
Time pressure
Time delay
Consent form
Public Goods Game under time constraint manipulation*
After the game:
• Belief regarding the contribution of other players
• Comprehension of the game
• Open question on why they made their decision
Player 1
Player 2
Trust Game
After the game:
• Comprehension of the game
• Open question on why they made their decision
Other trust measures
• Propensity to Trust Survey and filler items
• World Value Survey Trust and filler items
Other measures
• Experience with similar studies and research participation
• Social capital
• Perceived Awareness of the Research Hypothesis scale and other questions about demand effects
Demographics
• Age
• Gender
• Where they grew up
• Education
• Socioeconomic status
• Subjective socioeconomic status
• How many people in the room they knew
* Order of Public Goods Game and Trust Game was randomized
