# Supplementary material for: Correction: The role of trust in the social heuristics hypothesis
Source: PLoS One. 2021 Jan 27;16(1):e0241069. doi: 10.1371/journal.pone.0241069 (PMC7840021; doi:10.1371/journal.pone.0241069)
Supplement: S1 Table — (DOCX) [file pone.0241069.s001.docx]

**S1 Table. Regression tables.**

**Table A. Public goods game contributions predicted by high trust and time pressure with and without interaction and exclusions (Study 1).**

|  | | | | | | |
| --- | --- | --- | --- | --- | --- | --- |
|  | Dependent variable: | | | | | |
|  |  | | | | | |
|  | PGG contribution | | | | | |
|  | Full sample | | Excluding non-compliant | | Excluding non-comprehending | |
|  | (1) | (2) | (3) | (4) | (5) | (6) |
|  | | | | | | |
| High trust (HT) | 652.257^**^ | 670.037 | 466.745 | 569.895 | -1.451 | 769.236 |
|  | (317.252) | (452.047) | (417.907) | (472.467) | (433.499) | (626.397) |
|  |  |  |  |  |  |  |
| Time pressure (TP) | -117.516 | -99.737 | 1,798.635^***^ | 2,086.730^***^ | 28.118 | 791.842 |
|  | (317.252) | (452.047) | (499.873) | (789.595) | (433.700) | (623.561) |
|  |  |  |  |  |  |  |
| HT × TP |  | -35.157 |  | -482.342 |  | -1,463.296^*^ |
|  |  | (635.662) |  | (1,021.678) |  | (863.131) |
|  |  |  |  |  |  |  |
| Constant | 4,824.047^***^ | 4,814.657^***^ | 4,664.794^***^ | 4,612.413^***^ | 5,084.277^***^ | 4,633.875^***^ |
|  | (280.754) | (328.526) | (317.114) | (336.684) | (400.763) | (478.863) |
|  |  |  |  |  |  |  |
|  | | | | | | |
| Observations | 287 | 287 | 165 | 165 | 161 | 161 |
| R^2^ | 0.015 | 0.015 | 0.085 | 0.087 | 0.00003 | 0.018 |
| Adjusted R^2^ | 0.008 | 0.005 | 0.074 | 0.069 | -0.013 | -0.001 |
| Residual Std. Error | 2,684.376 (df = 284) | 2,689.100 (df = 283) | 2,665.933 (df = 162) | 2,672.350 (df = 161) | 2,724.874 (df = 158) | 2,708.856 (df = 157) |
| F Statistic | 2.221 (df = 2; 284) | 1.477 (df = 3; 283) | 7.548^***^ (df = 2; 162) | 5.082^***^ (df = 3; 161) | 0.002 (df = 2; 158) | 0.960 (df = 3; 157) |
|  | | | | | | |
| Note:  ^*^p<0.1; ^**^p<0.05; ^***^p<0.01  Standard errors are reported in parentheses | | | | | | |

**Table B. Public goods game contributions predicted by high trust, time pressure, and Faith in Intuition with and without interactions and exclusions (Study 1).**

|  | | | | | | |
| --- | --- | --- | --- | --- | --- | --- |
|  | Dependent variable: | | | | | |
|  |  | | | | | |
|  | PGG contribution | | | | | |
|  | Full sample | | Excluding non-compliant | | Excluding non-comprehending | |
|  | (1) | (2) | (3) | (4) | (5) | (6) |
|  | | | | | | |
| High trust (HT) | 607.813^*^ | 2,309.765 | 438.281 | 1,941.931 | -31.950 | 3,065.703 |
|  | (314.811) | (1,724.309) | (414.772) | (2,117.860) | (430.612) | (2,548.743) |
|  |  |  |  |  |  |  |
| Time pressure (TP) | -130.849 | 1,386.840 | 1,795.286^***^ | 5,372.518^**^ | -13.549 | 2,335.621 |
|  | (314.360) | (1,708.237) | (495.809) | (2,583.190) | (431.089) | (2,554.198) |
|  |  |  |  |  |  |  |
| Faith in Intuition (FI) | 614.036^**^ | 1,049.857^**^ | 582.159^*^ | 1,000.582^**^ | 653.740^*^ | 1,236.527^*^ |
|  | (244.025) | (416.460) | (303.918) | (462.394) | (356.372) | (678.101) |
|  |  |  |  |  |  |  |
| HT × TP |  | -17.214 |  | -427.875 |  | -1,344.068 |
|  |  | (631.421) |  | (1,013.444) |  | (862.546) |
|  |  |  |  |  |  |  |
| HT × FI |  | -497.943 |  | -423.874 |  | -700.791 |
|  |  | (493.365) |  | (615.412) |  | (732.021) |
|  |  |  |  |  |  |  |
| TP × FI |  | -443.868 |  | -988.731 |  | -485.080 |
|  |  | (492.168) |  | (732.968) |  | (731.638) |
|  |  |  |  |  |  |  |
| Constant | 2,774.502^***^ | 1,298.418 | 2,725.376^**^ | 1,286.669 | 2,882.779^**^ | 499.239 |
|  | (860.699) | (1,432.345) | (1,060.209) | (1,572.756) | (1,264.307) | (2,317.005) |
|  |  |  |  |  |  |  |
|  | | | | | | |
| Observations | 287 | 287 | 165 | 165 | 161 | 161 |
| R^2^ | 0.037 | 0.043 | 0.106 | 0.118 | 0.021 | 0.045 |
| Adjusted R^2^ | 0.027 | 0.022 | 0.089 | 0.085 | 0.002 | 0.008 |
| Residual Std. Error | 2,659.528 (df = 283) | 2,665.713 (df = 280) | 2,644.238 (df = 161) | 2,650.095 (df = 158) | 2,704.706 (df = 157) | 2,697.605 (df = 154) |
| F Statistic | 3.619^**^ (df = 3; 283) | 2.083^*^ (df = 6; 280) | 6.338^***^ (df = 3; 161) | 3.536^***^ (df = 6; 158) | 1.123 (df = 3; 157) | 1.202 (df = 6; 154) |
|  | | | | | | |
| Note: ^*^p<0.1; ^**^p<0.05; ^***^p<0.01  Standard errors are reported in parentheses | | | | | | |

**Table C. Public goods game contributions predicted by high trust, time pressure, and Need for Cognition with and without interactions and exclusions (Study 1).**

|  | | | | | | |
| --- | --- | --- | --- | --- | --- | --- |
|  | Dependent variable: | | | | | |
|  |  | | | | | |
|  | PGG contribution | | | | | |
|  | Full sample | | Excluding non-compliant | | Excluding non-comprehending | |
|  | (1) | (2) | (3) | (4) | (5) | (6) |
|  | | | | | | |
| High trust (HT) | 669.843^**^ | -655.854 | 466.152 | -132.746 | 7.566 | -1,900.215 |
|  | (316.983) | (1,957.657) | (419.038) | (2,400.053) | (434.430) | (2,587.027) |
|  |  |  |  |  |  |  |
| Time pressure (TP) | -90.988 | -1,441.011 | 1,793.403^***^ | -314.242 | 23.230 | -4,794.978^*^ |
|  | (317.305) | (2,001.048) | (501.435) | (3,542.212) | (434.491) | (2,627.873) |
|  |  |  |  |  |  |  |
| Need for Cognition (NFC) | 335.347 | 51.091 | 107.175 | -66.037 | 226.476 | -817.020 |
|  | (241.070) | (377.756) | (298.647) | (416.513) | (330.943) | (565.190) |
|  |  |  |  |  |  |  |
| HT × TP |  | 89.908 |  | -419.870 |  | -1,350.140 |
|  |  | (640.211) |  | (1,034.097) |  | (862.906) |
|  |  |  |  |  |  |  |
| HT × NFC |  | 336.406 |  | 181.606 |  | 721.079 |
|  |  | (487.163) |  | (608.397) |  | (664.844) |
|  |  |  |  |  |  |  |
| TP × NFC |  | 342.989 |  | 604.735 |  | 1,469.761^**^ |
|  |  | (500.185) |  | (869.255) |  | (669.907) |
|  |  |  |  |  |  |  |
| Constant | 3,504.072^***^ | 4,616.392^***^ | 4,250.965^***^ | 4,866.288^***^ | 4,220.163^***^ | 7,672.169^***^ |
|  | (989.420) | (1,502.295) | (1,196.189) | (1,636.769) | (1,324.982) | (2,154.783) |
|  |  |  |  |  |  |  |
|  | | | | | | |
| Observations | 287 | 287 | 165 | 165 | 161 | 161 |
| R^2^ | 0.022 | 0.026 | 0.086 | 0.091 | 0.003 | 0.053 |
| Adjusted R^2^ | 0.012 | 0.005 | 0.069 | 0.056 | -0.016 | 0.016 |
| Residual Std. Error | 2,679.968 (df = 283) | 2,689.580 (df = 280) | 2,673.130 (df = 161) | 2,691.339 (df = 158) | 2,729.470 (df = 157) | 2,686.426 (df = 154) |
| F Statistic | 2.131^*^ (df = 3; 283) | 1.221 (df = 6; 280) | 5.048^***^ (df = 3; 161) | 2.628^**^ (df = 6; 158) | 0.158 (df = 3; 157) | 1.427 (df = 6; 154) |
|  | | | | | | |
| Note: ^*^p<0.1; ^**^p<0.05; ^***^p<0.01  Standard errors are reported in parentheses | | | | | | |

**Table D. Public goods game contributions predicted by time pressure with and without exclusions (Study 1).**

|  | | | |
| --- | --- | --- | --- |
|  | Dependent variable: | | |
|  |  | | |
|  | PGG contribution | | |
|  | Full sample | Excluding non-compliant | Excluding non-comprehending |
|  | (1) | (2) | (3) |
|  | | | |
| Time pressure | -147.136 | 1,851.755^***^ | 28.310 |
|  | (318.714) | (497.983) | (428.553) |
|  |  |  |  |
| Constant | 5,168.549^***^ | 4,901.812^***^ | 5,083.429^***^ |
|  | (226.540) | (235.816) | (309.550) |
|  |  |  |  |
|  | | | |
| Observations | 287 | 165 | 161 |
| R^2^ | 0.001 | 0.078 | 0.00003 |
| Adjusted R^2^ | -0.003 | 0.073 | -0.006 |
| Residual Std. Error | 2,699.531 (df = 285) | 2,667.955 (df = 163) | 2,716.292 (df = 159) |
| F Statistic | 0.213 (df = 1; 285) | 13.827^***^ (df = 1; 163) | 0.004 (df = 1; 159) |
|  | | | |
| Note:  ^*^p<0.1; ^**^p<0.05; ^***^p<0.01  Standard errors are reported in parentheses | | | |

**Table E. Public goods game contributions predicted by high trust and intuition with and without interaction and exclusions (Study 2).**

|  | | | | | | |
| --- | --- | --- | --- | --- | --- | --- |
|  | Dependent variable: | | | | | |
|  |  | | | | | |
|  | PGG contribution | | | | | |
|  | Full sample | | Excluding experienced | | Excluding non-comprehending | |
|  | (1) | (2) | (3) | (4) | (5) | (6) |
|  | | | | | | |
| High trust (HT) | 0.688^***^ | 0.513 | 0.797^**^ | 0.370 | 1.104^***^ | 0.952^*^ |
|  | (0.247) | (0.346) | (0.371) | (0.517) | (0.383) | (0.526) |
|  |  |  |  |  |  |  |
| Intuition (INT) | 0.214 | 0.036 | 0.517 | 0.067 | 0.043 | -0.126 |
|  | (0.247) | (0.349) | (0.371) | (0.531) | (0.384) | (0.553) |
|  |  |  |  |  |  |  |
| HT × INT |  | 0.356 |  | 0.879 |  | 0.326 |
|  |  | (0.493) |  | (0.742) |  | (0.770) |
|  |  |  |  |  |  |  |
| Constant | 6.877^***^ | 6.964^***^ | 6.842^***^ | 7.054^***^ | 6.677^***^ | 6.758^***^ |
|  | (0.213) | (0.245) | (0.318) | (0.365) | (0.332) | (0.383) |
|  |  |  |  |  |  |  |
|  | | | | | | |
| Observations | 778 | 778 | 360 | 360 | 378 | 378 |
| R^2^ | 0.011 | 0.012 | 0.018 | 0.022 | 0.022 | 0.022 |
| Adjusted R^2^ | 0.008 | 0.008 | 0.013 | 0.014 | 0.016 | 0.014 |
| Residual Std. Error | 3.438 (df = 775) | 3.439 (df = 774) | 3.520 (df = 357) | 3.518 (df = 356) | 3.724 (df = 375) | 3.728 (df = 374) |
| F Statistic | 4.262^**^ (df = 2; 775) | 3.014^**^ (df = 3; 774) | 3.359^**^ (df = 2; 357) | 2.709^**^ (df = 3; 356) | 4.146^**^ (df = 2; 375) | 2.818^**^ (df = 3; 374) |
|  | | | | | | |
| Note: ^*^p<0.1; ^**^p<0.05; ^***^p<0.01  Standard errors are reported in parentheses | | | | | | |

**Table F. Public goods game contributions predicted by intuition with and without exclusions (Study 2).**

|  | | | |
| --- | --- | --- | --- |
|  | Dependent variable: | | |
|  |  | | |
|  | PGG contribution | | |
|  | Full sample | Excluding experienced | Excluding non-comprehending |
|  | (1) | (2) | (3) |
|  | | | |
| Intuition | 0.212 | 0.539 | 0.016 |
|  | (0.248) | (0.373) | (0.388) |
|  |  |  |  |
| Constant | 7.222^***^ | 7.238^***^ | 7.262^***^ |
|  | (0.174) | (0.260) | (0.265) |
|  |  |  |  |
|  | | | |
| Observations | 778 | 360 | 378 |
| R^2^ | 0.001 | 0.006 | 0.00000 |
| Adjusted R^2^ | -0.0003 | 0.003 | -0.003 |
| Residual Std. Error | 3.453 (df = 776) | 3.537 (df = 358) | 3.760 (df = 376) |
| F Statistic | 0.735 (df = 1; 776) | 2.090 (df = 1; 358) | 0.002 (df = 1; 376) |
|  | | | |
| Note: ^*^p<0.1; ^**^p<0.05; ^***^p<0.01  Standard errors are reported in parentheses | | | |
