# Supplementary material for: Correction: The role of trust in the social heuristics hypothesis
Source: PLoS One. 2021 Jan 27;16(1):e0241069. doi: 10.1371/journal.pone.0241069 (PMC7840021; doi:10.1371/journal.pone.0241069)
Supplement: S2 Table — (DOCX) [file pone.0241069.s002.docx]

**S2 Table. Estimated number of Study 1 participants per session.**

| Number of participants in session | Frequency |
| --- | --- |
| 5 | 1 |
| 6 | 1 |
| 7 | 1 |
| 8 | 26 |
| 10 | 1 |
| 11 | 2 |
| 12 | 3 |
| Total | 35 |
