# Supplementary material for: Correction: The role of trust in the social heuristics hypothesis
Source: PLoS One. 2021 Jan 27;16(1):e0241069. doi: 10.1371/journal.pone.0241069 (PMC7840021; doi:10.1371/journal.pone.0241069)
Supplement: S2 Fig — (DOCX) [file pone.0241069.s004.docx]

**S2 Fig. Histograms and plots.**

**Fig A. Distribution of decision times (left) and distribution of log10 decision times (right; Study 1).**

**Fig B. Unstandardized coefficients and 95% confidence intervals of public goods game contributions predicted by high trust, time pressure, and Faith in Intuition with and without interactions and exclusions (Study 1).**

Coefficients of regression with main effects are presented on the left and coefficients of regression with interactions and simple effects are presented on the right (simple effects are not included in the plot).

**Fig C. Unstandardized coefficients and 95% confidence intervals of public goods game contributions predicted by high trust, time pressure, and Need for Cognition with and without interactions and exclusions (Study 1).**

Coefficients of regression with main effects are presented on the left and coefficients of regression with interactions and simple effects are presented on the right (simple effects are not included in the plot).

**Fig D. Equivalence test for effect of time pressure on public goods game contributions (Study 1).**

**Fig E. Distribution of decision times (left) and distribution of log10 decision times (right; Study 2).**

**Fig F. Equivalence test for effect of intuition on public goods game contributions (Study 2).**
